# Digital transformation and marketing: a systematic and thematic literature review

**DOI:** 10.1007/s43039-023-00067-2

**Published:** 2023-03-15

**Authors:** Marco Cioppi, Ilaria Curina, Barbara Francioni, Elisabetta Savelli

**Affiliations:** 1 Department of Communication Sciences, Humanities and International Studies, University of Urbino Carlo Bo, Via Saffi 15, 61029 Urbino, Italy; 2 Department of Economics, Society, Politics, University of Urbino Carlo Bo, Via Saffi, 42, 61029 Urbino, Italy

**Keywords:** Digital transformation, Marketing, Systematic literature review, Thematic literature review, Synergistic framework

## Abstract

This article provides a systematic review of the extensive and fragmented literature focused on Digital Transformation (DT) and marketing by identifying the main themes and perspectives (i.e., employees, customers, and business processes) studied by previous research. By mapping the DT literature in the area of marketing, 117 articles, published between 2014 and 2020, have been identified. Through the adoption of a content analysis process, a multi-dimensional framework synthesizing the DT and marketing binomial has been provided. Results identify two thematical patterns: the macro-themes, related to the main digital technologies adopted within the marketing function, and the micro-themes, related to the effect/impact of these technologies on marketing processes and activities. Concerning the micro-themes, findings show how they have mainly studied from the customer and business processes’ perspectives, thus identifying an interesting research gap related to the analysis of the DT-marketing phenomenon from the employees’ standpoint. Based on these results, the paper derives a research agenda by also providing theoretical and managerial implications. Theoretically, it is the first systematic and thematic review focused on DT and marketing. In particular, it analyses this binomial from a broad and comprehensive perspective, thus offering a synergistic framework of the existing literature, which allows an inclusive vision and understanding about the phenomenon. At the managerial level, the paper could help organizations to enhance their awareness about marketing areas and processes that could better benefit from digitalization, thus driving the overall transition of firms towards DT.

## Introduction and background

Over the last decades, digital transformation (DT) has received growing attention in the business literature since it represents a prominent feature for organizations to be leaders of change and competitive in their domain (Kraus et al., 2022). At once, in light of the COVID-19 pandemic, the DT phenomenon has experienced an abrupt acceleration (Priyono et al., 2020), as firms and organizations are forced to redesign their strategies and operating models through a massive adoption of technologies in order to respond to the crisis-caused changes (Hai et al., 2021; Hanelt et al., 2021). Therefore, the necessity of analysing the DT topic has become ever more crucial in the last few years.

Conceptually, DT refers to all changes that digital technologies can bring in a firm’s business model, concerning products, processes, and organizational structures (Hess et al., 2016). Starting from this definition, it appears clear the pervasiveness of this phenomenon, which represents a real transition toward a new reality made of risks and challenges (Horvat and Szabo, 2019; Kraus et al., 2022; Vial, 2019). DT, indeed, can change every aspect of business, especially the marketing one (Caliskan et al., 2020).

Notably, the connection between DT and marketing has become ever more decisive in the last two years. The critical changes related to the COVID-19 crisis have particularly altered the firm and consumer relations, forcing companies to modify their marketing strategies through the massive exploitation of the digital technologies. In particular, marketing currently represents one of the main functions requiring to be adapted to the DT in order to protect firms’ competitiveness (Caliskan et al., 2020). By following this research stream, some authors have tried to synthetize the main impacts of DT on marketing practices (Shkurupskaya and Litovchenko, 2016; Sunday and Vera, 2018), including (i) The increasing spread of information and communication technology (ICT) in the marketing communication channels; (ii) The opportunity to adopt real-time communication with customers; (iii) The development of new relationships between producers and consumers; (iv) The increasing effectiveness of the marketing activities through the monitoring of real-time data. Meanwhile, other authors have specifically focused their attention on the main digital technologies able to offer significant benefits to the marketing function (Ardito et al., 2019; Cluley et al., 2019; Giannakis et al., 2019; Ungerman et al., 2018) by also categorizing them on the basis of the marketing mix (Caliskan et al., 2020).

Despite the DT-marketing topic has received growing attention, to date, no systematic review exists concerning the analysis of the DT phenomenon with specific application to the marketing processes and activities. Notably, several studies have tried to review the DT literature from very restricted research areas (Hanelt et al., 2021) different with respect to the broader one of marketing, such as B2B relationships (Hofacker et al., 2020), business model innovation (Favoretto et al., 2022; Li, 2020), accounting (Knudsen, 2020), multinational enterprises (George and Schillebeeckx, 2022), leadership (Carvalho et al., 2022; Henderikx and Stoffers, 2022), quality management (Dias et al., 2021; Thekkoote, 2022), production applications (D’Almeida et al., 2022), business management adaptability (Zhang et al., 2021), stakeholder management (Prebanić and Vukomanović, 2021), and sustainability (Gomez-Trujillo and Gonzalez-Perez, 2021). Faced with this context, some authors have tried to analyse and systematize the previous DT literature within broader research areas such as the business and management (Kraus et al., 2022) and the organizational change (Hanelt et al., 2021). However, despite these contributions, until now, no study has focused on reviewing the literature dedicated to the binomial DT-marketing.

Starting from these assumptions, the present study aims to provide a comprehensive review of the extant literature focused on DT in the marketing area by identifying the main themes and perspectives of analysis. More in detail, the paper addresses the following research questions: (i) What themes have been studied by previous research on DT in the field of marketing? (ii) What are the main perspectives adopted by the research on DT in the field of marketing?

To answer these research questions, the study has been organized in two phases: while in the first one the DT literature has been mapped by focusing on all studies addressing the digital transformation and marketing topics during the period 2014–2020, in the second phase a synergistic framework with the main macro and micro themes characterizing DT in the marketing area (concerning the digital technologies use and effects, respectively), along with the related analysed perspectives, has been provided.

By doing so, this study informs the academicians about the recent evolution of DT literature on marketing-related topics. Additionally, by proposing a synergistic framework of results, the paper provides a solid support for discussing and delineating future research directions. Finally, the main results of this review could help organizations to increase their awareness about marketing areas and processes that could better benefit from digitalization, thus driving the overall transition of firms towards DT.

The remainder of the paper is structured as follows. Section 2 presents the methodology and Sect. 3 outlines the descriptive and thematic results of the study. Section 4 provides theoretical and managerial implications and proposes future research directions based on the main gaps in existing literature. Finally, Sect. 5 concludes the study by also discussing the main limitations.

## Methodology

This study adopts the systematic review method (Tranfield et al., 2003) to detect, classify, and interpret “all the available research relevant to a particular research question, or topic area or phenomenon of interest” (Kitchenham, 2004; p. 1). Structurally, the review process has been divided into three phases: (i) Data collection; (ii) Paper selection; (iii) Content analysis.

The identification of specific keywords and terms represents the first systematic review step (Tranfield et al., 2003). In our research, the following string has been adopted: [“Digital transformation” AND “marketing”], with the final aim of identifying all the contributions simultaneously focused on these two topics, regardless of the subject area (e.g., business, management, etc.) and research approach (e.g., qualitative *vs*. quantitative). The Scopus database has been employed as it represents the broader abstract and citation database of peer-review literature, and it also contains most of the publications from other databases (Guerrero et al., 2015).

All the proposed document typologies have been included in the analysis (i.e., articles, conference papers, conference reviews, literature reviews) by applying the above string on their title, abstract, and keywords (Table 1). As for the time frame, contributions published between 2014 and 2020 have been considered following the study of Vaska and Colleagues (2021), which reveals a growth in interest toward DT field, particularly from 2014.

**Table 1:** Literature review’s selection criteria

	Selection criteria
Keyword	“Digital transformation” AND “marketing”
Database	Scopus
Subject areas	All
Source	Article title; Abstract; Keyword
Time frame	2014–2020
Document typology	Article; Conference paper; Conference review; Literature review

**Fig. 1:**
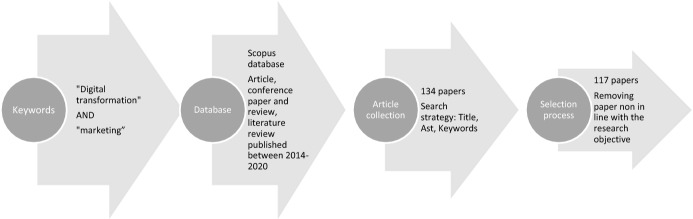
Main steps of the literature analysis

A total number of 134 publications have been identified and further selected by considering only those studies effectively focused on the investigated topics. At the end of this process, 117 documents have been retained and subjected to content analysis to identify the main DT themes and perspectives in the marketing field (Fig. 1).

Notably, the content analysis allows the “systematic and theory-guided reduction of a large amount of text data from any type of communication down to its essence by classifying the material into unifying categories” (Hanelt et al., 2021; p. 1163). It is distinguished from other qualitative procedures, such as the thematic one, since it permits to build category systems in line with the research questions, thus providing both qualitative and quantitative insights (Mikelsone et al., 2019).

## Results and discussion

In the following sub-paragraphs, the descriptive and thematic results of the literature review will be presented.

### Descriptive results

**Fig. 2:**
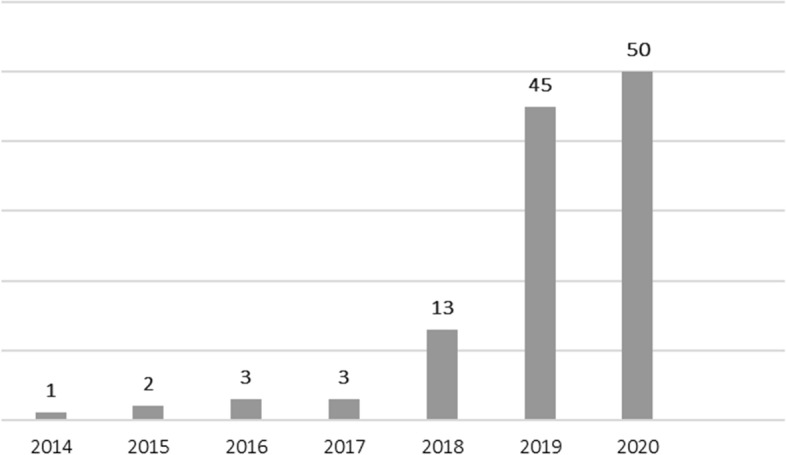
Year distribution of contributions

Concerning the yearly research trend (Fig. 2), a growing interest in the digital transformation-marketing topic emerged during the time-period under review. Particularly, we went from only one contribution published in 2014 to three in 2017; starting from 2018, the attention increased with 13 published articles, while the most significant peaks have been reached between 2019 and 2020, characterized by the higher production of contributions (45 in 2019 and 50 in 2020).

Table 2 ranks the sources with the highest number of published contributions focused on the investigated topic. Ninety-three sources have published the 117 reviewed papers with the more relevant contribution from the Advances in Intelligent Systems and Computing (3,4%), followed by Industrial Marketing Management (3,4%), and IOP Conferences series: Materials Science and Engineering (3,4%), Communications in Computer and Information Science (2,6%), and Journal of Physics (2,6%).

**Table 2:** Source distribution per reviewed contributions

Sources	Source type	Number of papers	Authors
Advances in Intelligent Systems and Computing	Book series	4	Polyakov and Gordeeva (2020), Rolbina et al. (2020), Venermo et al. (2020), Hahn (2019),
Industrial Marketing Management	Journal	4	Endres et al. (2020), Sivarajah et al. (2020), Taylor et al. (2020), Handfield (2019)
IOP Conference Series: Materials Science and Engineering	Conference Proceedings	4	Bekmurzaev et al. (2020), Pirogova et al. (2020), Ianenko et al. (2019), Voronkova (2018)
Communications in Computer and Information Science	Book series	3	Del Giorgio and Mon (2019), Hsu et al. (2019), Majumder et al. (2018)
Journal of Physics: Conference Series	Conference Proceedings	3	Cahyadi (2020), Li et al. (2020), Lin et al. (2020),
Applied Marketing Analytics	Journal	2	Bughin et al. (2019), Subramani (2019)
IEEE Engineering Management Review	Journal	2	Almeida et al. (2020), Kim (2020),
IEEE Access	Journal	2	Al-Azani and El-Alfy (2020), Miklosik and Evans (2020)
Journal of Brand Strategy	Journal	2	Dasser (2019), Lieberman (2019)
Profesional de la Informacion	Journal	2	Álvarez-Flores et al. (2018), Serrano-Cobos (2016)
Lecture Notes in Networks and Systems	Book series	2	Agafonova et al. (2020), Kazaishvili and Khmiadashvili (2020),
Lecture Notes in Computer Science	Book series	2	Alassani and Göretz (2019), Van Osch et al. (2019)
Lecture Notes in Business Information Processing	Book series	2	Graf et al. (2019), Muñoz and Avila (2019)
Smart Innovation, Systems and Technologies	Book series	2	Azeredo et al. (2020), Chehri and Jeon (2019),
ACM International Conference Proceeding Series	Conference Proceedings	2	Kuimov et al. (2019), Anh et al. (2019)
eLearning and Software for Education Conference	Conference Proceedings	2	Paraschiveanu et al. (2020), Minculete and Minculete (2019)
Subtotal		**40**	

Additional sources with only one published contribution are shown in Table 3. Notably, fifty-seven sources are Journals, eighteen are conference proceedings, and two sources are book series. Concerning the Journals, those from a domain especially related to the business management, society, technology innovation, economics, and engineering have shown interest toward this specific issue. With respect to the conference proceedings, the main fields of study concern the smart trends, technology innovation management, computer science, and information systems. Finally, regarding the book series, they are specifically focused on the information and communication and tourism research streams.

**Table 3:** Additional sources (with only one published contribution)

Source type	Number of sources	Field of studies
Journal	57	Business management (12); Society (7); Technology innovation (6); Economics (6); Engineering (6); Marketing (3); Information systems (2); Digital and social media marketing (2); Environmental science (2); Business environment (2); Product development (2); Small businesses (1) Strategy and management (1); Computers and communication (1); Advanced science and technology (1); Managerial science (1); Medical sector (1); Quality assurance (1)
Conference Proceedings	18	Smart trends (2); Technology Innovation Management (2); Computer science (2); Information systems (2); Information, Communication and Electronic Technology (1); Society (1); Tourism (1); Environmental Science (1); Offshore Technology (1); Customer experience (1); Artificial Intelligence, application, and innovations (1); International Business Information Management (1); Enterprise computing (1) Engineering (1)
Book series	2	Information and communication technology (1); Tourism (1)
Subtotal	**77**	

The source’s distribution is informant about the main future publication opportunities in the area of DT and marketing. Equally relevant is the result related to the contributions’ ranking per citation since it allows to figure out the widespread and dissemination of the analysed research stream. Table 4 shows the top-ten contributions in terms of citations. Notably, the more cited contributions are very recent (published between 2019 and 2020) and mainly focused on the following topics: technological innovations as enablers for firms’ digitalization strategies (Ballestar et al., 2019; Gil-Gomez et al., 2020; Hausberg et al., 2019; Peter et al., 2020; Sestino et al., 2020; Ulas, 2019; Yigitcanlar et al., 2020) and business sustainability (Sivarajah et al., 2020), and the impact of the COVID-19 crisis on consumers’ (Kim, 2020) and firms’ digital behaviours (Almeida et al., 2020).

**Table 4:** Ranking of contributions per citations (Top-ten)

Title contribution	Source	Number of citations	Author/s
The impact of COVID-19 on consumers: Preparing for digital sales	IEEE Engineering Management Review	135	Kim (2020)
Internet of Things and Big Data as enablers for business digitalization strategies	Technovation	91	Sestino et al. (2020)
Role of big data and social media analytics for business to business sustainability: A participatory web context	Industrial Marketing Management	78	Sivarajah et al. (2020)
The challenges and opportunities in the digitalization of companies in a Post-COVID-19 World	IEEE Engineering Management Review	72	Almeida et al. (2020)
Digital transformation process and SMEs	Procedia Computer Science	63	Ulas (2019)
Customer relationship management: digital transformation and sustainable business model innovation	Economic Research-Ekonomska Istrazivanja	51	Gil-Gomez et al. (2020)
Research streams on digital transformation from a holistic business perspective: a systematic literature review and citation network analysis	Journal of Business Economics	47	Hausberg et al. (2019)
Strategic action fields of digital transformation: An exploration of the strategic action fields of Swiss SMEs and large enterprises	Journal of Strategy and Management	46	Peter et al. (2020)
Artificial intelligence technologies and related urban planning and development concepts: How are they perceived and utilized in Australia?	Journal of Open Innovation	40	Yigitcanlar et al. (2020)
Predicting customer quality in e-commerce social networks: a machine learning approach	Review of Managerial Science	32	Ballestar et al. (2019)

Finally, concerning the adopted methodologies, 93 (79,5%) contributions are based on qualitative methods, while the remaining 24 (20,5%) are quantitative in nature.

### Thematic results

By employing the content analysis, it has been possible to extract the main DT themes and perspectives in the marketing fields. As for the DT themes, two main clusters have been identified:

1. Macro-themes related to the use of digital technologies within the marketing function;
2. Micro-themes related to the effects emerging from the use of digital technologies on marketing processes and activities.

#### Macro-themes related to the use of digital technologies

The identification of the most investigated digital technologies analysed in the marketing domain by the reviewed contributions represents the first result deriving from the content analysis. Appendix 1 displays the list of technologies along with their main conceptualizations. As shown in Table 5, the majority of contributions (67,1%) have focused their attention on the analysis of specific digital tools. In particular, the social media channels (social media marketing) represent the most examined technology (being investigated by 9,4% of the selected studies), followed by Big Data (8,7%), mobile marketing (i.e., mobile technology and smart apps) (8,1%), Internet of Things (6,7%), Artificial Intelligence (6,7%), and Industry 4.0 (6,7%). The remaining technologies (i.e., Machine learning; Online collaborative/support platforms/systems; Virtual/Augmented Reality; Websites/SEO; Cloud infrastructures; Chatbots; Drones/Smart robots; Security Protection systems; 3D print) have experienced a reduced interest by the extant literature (less than 6% of the identified contributions). Finally, a not negligible percentage of studies (32,9%) has analysed the topic of digitalization without investigating specific technologies. Rather, they broadly referred to the “digitalization phenomenon” as an overall macro-theme investing the marketing area.

**Table 5:** Macro-themes (Analysed digital technologies within the marketing function)

Macro-themes	Frequency (No.)	Frequency (%)	Author/s
Social media channels/Social media marketing	14	9,4%	Al-Azani and El-Alfy (2020), Kazaishvili and Khmiadashvili (2020), Kim (2020), Melović et al. (2020), Safiullin et al. (2020), Sivarajah et al. (2020), Yusmarni et al. (2020), Alassani and Göretz (2019), Hahn (2019), Kumar-Singh and Thirumoorthi (2019), Lestari et al. (2019), Rebelli (2019), Kaczorowska-Spychalska (2018), Majumder et al. (2018)
Big Data	13	8,7%	Almaslamani et al. (2020), Almeida et al. (2020), Miklosik and Evans (2020), Sestino et al. (2020), Sivarajah et al. (2020), Visan and Ciurea (2020), Sargut (2019), Bohnsack and Liesner (2019), Kumar-Singh and Thirumoorthi (2019), Ulas (2019), Zimand Sheiner and Earon (2019), Papagiannopoulus and Lopez (2018), Serrano-Cobos (2016)
Mobile marketing (Mobile technology, Smart apps)	12	8,1%	Attaran and Attaran (2020), Cahyadi (2020), Hamidi et al. (2020), Sundaram et al. (2020), Visan and Ciurea (2020), Garg et al. (2019), Hahn (2019), Kumar-Singh and Thirumoorthi (2019), Lekunze and Luvhengo (2019), Ulas (2019), Cherviakova and Cherviakova (2018), Kaczorowska-Spychalska (2018)
Internet of Things (IoT)	10	6,7%	Almeida et al. (2020), Hamidi et al. (2020), Li et al. (2020), Peter et al. (2020), Sestino et al. (2020), Voipio et al. (2020), Chehri and Jeon (2019), Kumar-Singh and Thirumoorthi (2019), Ulas (2019), Serrano-Cobos (2016)
Artificial Intelligence	10	6,7%	Almeida et al. (2020), Polyakov and Gordeeva (2020), Yigitcanlar et al. (2020), Sargut (2019), Subramani (2019), Ianenko et al. (2019), Kumar-Singh and Thirumoorthi (2019), Ulas (2019), Cherviakova and Cherviakova (2018), Serrano-Cobos (2016)
Industry 4.0 and 5C	10	6,7%	Bekmurzaev et al. (2020), Caliskan et al. (2020), Hamidi et al. (2020), Naglič et al. (2020), Yusmarni et al. (2020), Polyyakov and Gordeeva (2020), Chehri and Jeong (2019), Del Giorgio and Mon (2019), Nosalska and Mazurek (2019), Ulas (2019)
Machine Learning	6	4,0%	Kazaishvili and Khmiadashvili (2020), Miklosik and Evans, (2020), Polyakov and Gordeeva (2020), Ballestar et al., (2019), Sargut (2019), Subramani (2019),
Online collaborative/ support platforms/systems	5	3,4%	Azeredo et al. (2020), Bhatnagar and Grosse (2019), Minculete and Minculete (2019), Munz et al., (2019), Bruskin et al. (2017)
Virtual/ Augmented Reality	5	3,4%	Kim (2020), Hausberg et al. (2019), Kumar-Singh and Thirumoorthi (2019), Ulas (2019), Voronkova (2018)
Websites/SEO	4	2,7%	Natorina (2020), Yusmarni et al., (2020), Ballestar et al. (2019), García et al. (2019)
Cloud Infrastructure	3	2,0%	Visan and Ciurea (2020), Kumar-Singh and Thirumoorthi (2019), Ulas (2019)
Chatbots	3	2,0%	Damnjanovic (2019), Sargut (2019), Ulas (2019)
Drones/Smart Robots (Robotics)	2	1,3%	Almeida et al. (2020), Ulas (2019)
Security Protection systems	2	1,3%	Li et al. (2020), Munz et al. (2019)
3D Print	1	0,7%	Ulas (2019)
Digitalization phenomenon	49	32,9%	Agafonova et al. (2020), Andriole (2020), Bollweg et al. (2020), Calle et al. (2020), Dethine et al. (2020), Endres et al. (2020), Federico (2020), Gil-Gomez et al. (2020), Krasonikolakis et al. (2020), Lin et al. (2020), Oxoli et al. (2020), Pirogova et al. (2020), Rados et al. (2020), Rahimian (2020), Rolbina et al. (2020), Saravanabhavan et al. (2020), Shkarlet et al. (2020), Taylor et al. (2020), Vaganova et al. (2020), Venermo et al. (2020), Chantayarkul et al. (2019), Bughin et al. (2019), Dasser (2019), Di Gregorio et al. (2019), Fiodorov and Ochara (2019), Fokina and Barinov (2019), Graf et al. (2019), Handfield (2019), Hsu et al. (2019), Hughes and Vafeas (2019), Kuimov et al. (2019), Lieberman (2019), Nagano (2019), Saito and Nishio (2019), Yasynska et al. (2019), Álvarez-Flores et al. (2018), Barann (2018), Hafezieh and Pollock (2018), Heuchert et al. (2018), Persson et al. (2018), Ruggeri et al. (2018), Seitz and Burosch (2018), Andieva and Kapelyuhovskaya (2017), Kwon and Park (2017), Escadafal (2015), Van Belleghem (2015), Barnett (2014)
**Total**	**149**	**100,0%**	

The sum of the identified macro-themes (*n* = 149) exceeds the number of papers analysed during the review process (*n* = 117) since some papers have simultaneously examined more than one macro-theme.

#### Micro-themes related to the effects emerging from the use of digital technologies

The second result achieved by the content analysis concerns the main effects (i.e., micro-themes) deriving from the adoption and exploitation of the already identified digital technologies (Par. 3.2.1) on the marketing function. The most examined effects fall within the following areas: customer relationship management, customer connectivity, and customer centricity (12,3%), human resources (10,3%), digital metrics (8,8%), customer experience/journey (8,3%), business process efficiency (8,3%), MarTech (7,8%), market knowledge (7,4%), communication policy (5,9%), and customer behaviour (5,4%). The remaining effects (i.e., product policy, sales processes; production; buying/consumption processes; value co-creation; supply chain; branding; customer service; etc.) received less attention, being investigated by less than 5% of the identified contributions (Table 6).

**Table 6:** Micro-themes (Main effects deriving from the adoption and exploitation of the digital technologies)

Micro-themes (Adopted technologies’ effect)	Frequency (N.)	Frequency (%)	Author/s
Customer relationship management/customer connectivity/centricity	25	12,3%	Almaslamani et al. (2020), Cahyadi (2020), Caliskan et al. (2020), Gil-Gomez et al. (2020), Peter et al. (2020), Rolbina et al. (2020), Shkarlet et al. (2020), Sivarajah et al. (2020), Taylor et al. (2020), Ballestar et al. (2019), García et al. (2019), Graf et al. (2019), Hahn (2019), Handfield (2019), Hausberg et al. (2019), Ianenko et al. (2019), Kumar-Singh and Thirumoorthi (2019), Nosalska and Mazurek (2019), Subramani (2019), Barann (2018), Cherviakova and Cherviakova (2018), Papagiannopoulus and Lopez (2018), Serrano-Cobos (2016), Van Belleghem (2015)
Human resources (enhanced employees skills in using technology)	21	10,3%	Almeida et al. (2020), Andriole (2020), Attaran and Attaran (2020), Azeredo et al. (2020), Calle et al. (2020), Dethine et al., (2020), Peter et al. (2020), Rahimian (2020), Shkarlet et al. (2020), Bhatnagar and Grosse (2019), Dasser (2019), Di Gregorio et al. (2019), Fiodorov and Ochara (2019), Minculete and Minculete (2019), Subramani (2019), Ulas (2019), Zimand Sheiner and Earon (2019), Álvarez-Flores et al. (2018), Hafezieh and Pollock (2018), Know and Park (2017), Van Belleghem (2015)
Digital metrics	18	8,8%	Al-Azani and El-Alfy (2020), Almaslamani et al. (2020), Lin et al. (2020), Melovic et al. (2020), Rados et al. (2020), Safiullin et al. (2020), Saravanabhavan et al. (2020), Sivarajah et al. (2020), Bughin et al. (2019), Garg et al. (2019), Hausberg et al. (2019), Hsu et al. (2019), Lestari et al. (2019), Munz et al. (2019), Nagano (2019), Yasynska et al. (2019), Papagiannopoulus and Lopez (2018), Majumder et al. (2018)
Customer experience/journey	17	8,3%	Hamidi et al., (2020), Taylor et al. (2020), Venermo et al. (2020), Dasser (2019), Fokina and Barinov (2019), García et al. (2019), Kuimov et al. (2019), Kumar-Singh and Thirumoorthi (2019), Nosalska and Mazurek (2019), Lieberman (2019), Saito and Nishio (2019), Papagiannopoulus and Lopez (2018), Hafezieh and Pollock (2018), Heuchert et al. (2018), Persson et al. (2018), Escadafal (2015), Van Belleghem (2015)
Business process efficiency	17	8,3%	Bekmurzaev et al. (2020), Cahyadi (2020), Federico (2020), Li et al. (2020), Miklosik and Evans (2020), Natorina (2020), Sestino et al. (2020), Chantayarkul et al. (2019), Chehri and Jeong (2019), Fiodorov and Ochara (2019), Graf et al. (2019), Kuimov et al. (2019), Sargut (2019), Yasynska et al. (2019), Papagiannopoulus and Lopez (2018), Ruggieri et al. (2018), Bruskin et al. (2017)
MarTech (Marketing Technology)	16	7,8%	Almeida et al. (2020), Andriole (2020), Caliskan et al. (2020), Calle et al. (2020), Federico (2020), Krasonikolakis et al. (2020), Rahimian (2020), Shkarlet et al. (2020), Yigitcanlar et al. (2020), Chantayarkul et al. (2019), Lekunze and Luvhengo (2019), Barann (2018), Hafezieh and Pollock (2018), Kwon and Park (2017), Van Belleghem (2015)
Market knowledge	15	7,4%	Caliskan et al. (2020), Endres et al. (2020), Peter et al. (2020), Rados et al. (2020), Bohnsack and Liesner (2019), Garg et al. (2019), Hausberg et al. (2019), Hsu et al. (2019), Ianenko et al. (2019), Kuimov et al. (2019), Lestari et al. (2019), Munz et al. (2019), Nosalska and Mazurek (2019), Yasynska et al. (2019), Papagiannopoulus and Lopez (2018)
Communication policy	12	5,9%	Caliskan et al. (2020), Oxoli et al. (2020), Peter et al. (2020), Shkarlet et al. (2020), Yusmarni et al. (2020), Alassani and Goretz (2019), Ballestar et al. (2019), Nosalska and Mazurek (2019), Dasser (2019), Cherviakova and Cherviakova (2018), Kaczorowska-Spychalska (2018)
Customer behaviour	11	5,4%	Almaslamani et al. (2020), Rados et al. (2020), Del Giorgio and Mon (2019), Fokina and Barinov (2019), Hahn (2019), Ianenko et al. (2019), Kumar-Singh and Thirumoorthi (2019), Lieberman (2019), Rebelli (2019), Papagiannopoulus and Lopez (2018), Kaczorowska-Spychalska (2018)
Product policy	9	4,4%	Caliskan et al. (2020), Polyakov and Gordeeva (2020), Rados et al. (2020), Shkarlet et al. (2020), Voipio et al. (2020), Bohnsack and Liesner (2019), Ianenko et al. (2019), Nosalska and Mazurek (2019), Ulas (2019)
Sales processes	6	2,9%	Almeida et al. (2020), Venermo et al. (2020), Damnjanovic (2019), Dasser (2019), Kumar-Singh and Thirumoorthi (2019), Seitz and Burosch (2018)
Production processes	6	2,9%	Shkarlet et al. (2020), Vaganova et al. (2020), Chehri and Jeong (2019), Del Giorgio and Mon (2019), Nosalska and Mazurek (2019), Andieva and Kapelyuhovskaya (2017)
Buying/consumption processes	6	2,9%	Cahyadi (2020), Kim (2020), Venermo et al. (2020), Kumar-Singh and Thirumoorthi (2019), Voronkova (2018), Barnett (2014)
Value co-creation/value proposition	5	2,5%	Saravanabhavan et al. (2020), Taylor et al. (2020), Fokina and Barinov (2019), Hughes and Vafeas (2019), Kaczorowska-Spychalska (2018)
Supply chain processes	5	2,5%	Bekmurzaev et al. (2020), Safiullin et al. (2020), Sundaram et al. (2020), Voipio et al. (2020), Kumar-Singh and Thirumoorthi (2019)
Branding	4	2,0%	Kazaishvili and Khmiadashvili (2020), Melović et al. (2020), Natorina (2020), Rahimian (2020)
Customer service	3	1,5%	Lin et al. (2020), Safiullin et al. (2020), Lieberman (2019)
Export market orientation/export performance	2	1,0%	Dethine et al. (2020), Naglič et al. (2020)
Smart cities/factories	2	1,0%	Visan and Ciurea (2020), Chehri and Jeong (2019)
Drivers/barriers/risks of digitalization	2	1,0%	Bollweg et al. (2020), Pirogova et al. (2020)
Neuromarketing	1	0,5%	Polyyakov and Gordeeva (2020)
Social responsibility	1	0,5%	Agafonova et al. (2020)
**TOTAL**	204^*^	**100,0**	

The sum of the identified micro-themes (*n* = 204) exceeds the number of papers analysed during the review process (*n* = 117) since some papers have simultaneously examined more than one micro-theme.

The content analysis allowed as to go deep into the study of each micro-theme by revealing both a detailed list of specific sub-themes (Table 7) and the main perspectives of analysis adopted in the reviewed manuscripts (Table 8).

**Table 7:** Micro-themes and sub-themes

Micro-themes (Adopted technologies’ effect)	Sub-themes
Customer relationship management/customer connectivity/centricity	(i) Use of digital tools for interacting with B2B and B2C consumers
Human resources (enhanced employees skills in using technology)	(i) Enhanced employees skills in using technology; (ii) Most prominent job positions of the future (digital marketing manager; social media manager; big data/data analyst); (iii) Job offers on the internet and required knowledge and skills; (iv) Training of managers and marketers; (v) Continued professional development of the staff; (vi) The need for educational and training actions (Acquisition of new skills for integrating the digital channels into appropriate marketing activities)
Digital metrics	(i) Analysis of the digital results (through social media insights/google analytics); (ii) Social media monitoring/online monitoring tools; (iii) Data mining/sharing; (iv) Sentiment analysis
Customer experience/journey	(i) Evolution of the customers’/buyers’ experience and journey in the digital context; (ii) Customers’ online contact point
MarTech (Marketing Technology)	(i) Blending of technological tools (such as software platforms, systems, tools) with soft skills; (ii) Traditional/digital; (iii) The integration of physical and online platforms; (iv) Bricks and clicks; (v) Omni-channel approach; (vi) Balancing between automation and human interactions
Market knowledge	(i) Adoption of digital tools for obtaining market knowledge (benchmarking; market trends; market opportunities and threats)
Communication policy	(i) Content marketing strategies; (ii) Marketing automation; (iii) Recommendation-based digital marketing strategies; (iv) User generated content (User recruitment); (v) WOM; (vi) Influencer marketing
Customer behaviour	(i) Impact of digital tools on customers preferences/behaviours; (ii) Monitoring customers’ behaviours
Business process efficiency	(i) Operational/organizational excellence; (ii) Digital transformation and business process efficiency
Product policy	(i) Personalization and customization of products/services; (ii) Open innovation; (iii) Intelligent products/packaging; (iv) The effect of digital information sources in the development of information-based products and services; (v) Growth hacking (product development + digital marketing + data analysis)
Sales processes	(i) The impact of digital tools on the sales processes
Production processes	(i) Digital production systems/machines able to increase the process quality; (ii) The insertion of technologies in the automation and control of production processes
Value co-creation/value proposition	(i) Co-creation of value (with customers)
Buying/consumption processes	(i) The impact of digital tools on customers buying processes (online shopping); (ii) E-commerce; (iii) Structural change in consumption during COVID-19
Supply chain processes	(i) The use of digital technology in the supply chain processes
Branding	(i) Brand online visibility; (ii) Digital presence; (iii) Digital identity
Customer service	(i) The role of digital tools in the online customer service; (ii) Electronic services for assessing customer satisfaction
Export market orientation/export performance	(i) Market diversification and export performance of firms; (ii) The synergistic effects of market orientation, implementation, and internationalization on firm performance in the context of digital transformation
Smart cities/factories	(i) Smart cities/factories
Drivers/barriers/risks of digitalization	(i) The risk/barriers of digitalization (i.e., lack of awareness; privacy violation; lack of qualified personnel; distrust of citizens; the problem of training older employees; high implementation costs; Job cuts; (ii) Drivers of digitalization (i.e., increase firms’ competitiveness; reduce routine operations; create quality infrastructure; faster decision making and services; reduce the intermediaries’ numbers in the supply-chain processes)
Neuromarketing	(i) Artificial neural networks
Social responsibility	(i) Social responsibility implementation in the digital environment (directions of social responsibility implementation in the digital era are different; programs tend to be inconsistent and implemented within the societal marketing, value marketing, and traditional marketing concept. Moreover, firms tend not to perceive social responsibility as a noteworthy part of business activity in the digital environment)

**Table 8:** Micro-themes grouped by the analysed perspective

Micro-themes (freq.)	Analysed perspective (total freq.)
Human resources (21)	Employees Perspective (EP): 39 = 19,1%
MarTech (Marketing Technology) (16)	
Smart factories (2)	
Customer relationship management/customer connectivity/centricity (25)	Customer Perspective (CP): 68 = 33,3%
Customer experience/journey (17)	
Customer behaviour (11)	
Buying/consumption processes (6)	
Value co-creation/value proposition (5)	
Customer service (3)	
Neuromarketing (1)	
Digital metrics (18)	Business process perspective (BPP): 97 = 47,5%
Business process efficiency (17)	
Market knowledge (15)	
Communication policy (12)	
Product policy (9)	
Sales processes (6)	
Production processes (6)	
Supply chain processes (5)	
Branding (4)	
Export market orientation/export performance (2)	
Drivers/barriers/risks of digitalization (2)	
Social responsibility (1)	

Specifically, three main perspectives emerged from our study, namely employees, customers, and business. While the employee perspective focuses on the human resources and their coexistence with new technologies, the customer one is mainly related to the digital opportunities offered on the consumer side, especially concerning the overall shopping journey. Finally, the process-focused perspective is primarily concerned with the influence of digital technologies on the different business practices and procedures.

#### Macro-themes, micro-themes, and analysed perspectives: a combined overview

In this section, the macro-themes, micro-themes, and analysed perspectives will be combined with the final aim of building a comprehensive overview (Table 9).

**Table 9:** Macro-themes, micro-themes, and analysed perspectives: a combined overview

Macro-themes	Micro-themes	Analysed perspectives
Social media channels	/	*EP (/)*
	Customer behaviour (4) Customer relationship management/customer connectivity/centricity (3) Customer experience/journey (1) Buying/consumption process (2) Value co-creation/value proposition (1) Customer service (1)	*CP (12)*
	Digital metrics (6) Communication policy (3) Branding (2) Supply chain processes (2) Market knowledge (1) Sales processes (1)	*BPP (15)*
Big data	Human resources (3) MarTech (1) Smart factories (1)	*EP (5)*
	Customer relationship management/customer connectivity/centricity (5) Customer behaviour (3) Customer experience/journey (2) Buying/consumption process (1)	*CP (11)*
	Business process efficacy (4) Digital metrics (3) Market knowledge (2) Sales processes (2) Product policy (1) Supply chain processes (1)	*BPP (13)*
Mobile marketing	Human resources (2) MarTech (1) Smart factories (1)	*EP (4)*
	Customer relationship management/customer connectivity/centricity (4) Customer behaviour (3) Customer experience/Journey (2) Buying/consumption process (2) Value co-creation/value proposition (1)	*CP (12)*
	Communication policy (2) Supply chain processes (2) Digital metrics (1) Market knowledge (1) Business process efficacy (1) Product policy (1) Sales processes (1)	*BPP (9)*
Internet of things (IoT)	Human resources (3) MarTech (1) Smart factories (1)	*EP (5)*
	Customer relationship management/customer connectivity/centricity (3) Customer experience/journey (2) Customer behaviour (1) Buying/consumption process (1)	*CP (7)*
	Business process efficacy (3) Product policy (2) Sales processes (2) Supply chain processes (2) Market knowledge (1) Communication policy (1) Production processes (1)	*BPP (12)*
Artificial intelligence	Human resources (3) MarTech (2)	*EP (5)*
	Customer relationship management/customer connectivity/centricity (5) Customer behaviour (2) Customer experience/Journey (1) Buying/consumption process (1) Neuromarketing (1)	*CP (10)*
	Product policy (3) Sales processes (2) Communication policy (1) Market knowledge (1) Business process efficacy (1) Supply chain processes (1)	*BPP (9)*
Industry 4.0	Human resources (1) MarTech (1) Smart factories (1)	*EP (3)*
	Customer relationship management/customer connectivity/centricity (2) Customer experience/Journey (2) Customer behaviour (1) Neuromarketing (1)	*CP (6)*
	Product policy (4) Production processes (3) Communication policy (3) Market knowledge (2) Business process efficacy (2) Supply chain processes (1) Export market orientation/export performance (1)	*BPP (16)*
Machine learning	Human resources (1)	*EP (1)*
	Customer relationship management/customer connectivity/centricity (2) Neuromarketing (1)	*CP (3)*
	Business process efficacy (2) Communication policy (1) Product policy (1) Branding (1)	*BPP (5)*
Online collaborative/support platforms/systems	Human resources (3)	*EP (3)*
	/	*CP (/)*
	Digital metrics (1) Market knowledge (1) Business process efficiency (1)	*BPP (3)*
Virtual/Augmented Reality	Human resources (1)	*EP (1)*
	Buying/consumption process (3) Customer relationship management/customer connectivity/centricity (2) Customer experience/Journey (1) Customer behaviour (1)	*CP (7)*
	Digital metrics (1) Market knowledge (1) Sales processes (1) Product policy (1) Supply chain processes (1)	*BPP (5)*
Websites/ SEO	/	*EP (/)*
	Customer relationship management/customer connectivity/centricity (2) Customer experience/Journey (1)	*CP (3)*
	Communication policy (2) Branding (1) Business process efficiency (1)	*BPP (4)*
Cloud infrastructure	Human resources (1) Smart factories (1)	*EP (2)*
	Customer relationship management/customer connectivity/centricity (1) Customer experience/Journey (1) Customer behaviour (1) Buying/consumption process (1)	*CP (4)*
	Product policy (1) Sales processes (1) Supply chain processes (1)	*BPP (3)*
Chatbots	Human resources (1)	*EP (1)*
	/	*CP (/)*
	Business process efficiency (1) Product policy (1) Sales processes (1)	*BPP (3)*
Drones/Smart robots	Human resources (2) MarTech (1)	*EP (3)*
	/	*CP (/)*
	Product policy (1) Sales processes (1)	*BPP (2)*
Security protection systems	/	*EP (/)*
	/	*CP (/)*
	Digital metrics (1) Market knowledge (1) Business process efficiency (1)	*BPP (3)*
3D print	Human resources (1)	*EP (1)*
	/	*CP (/)*
	Product policy (1)	*BPP (1)*
Digitalization phenomenon	Human resources (12) MarcTech (12)	*EP (24)*
	Customer experience/Journey (12) Customer relationship management/customer connectivity/centricity (9) Value co-creation/value proposition (4) Customer behaviour (3) Customer service (2) Buying/consumption process (2)	*CP (32)*
	Digital metrics (7) Business process efficacy (7) Market knowledge (5) Communication policy (4) Sales processes (3) Production processes (3) Product policy (2) Drivers/barriers/risk of digitalization (2) Branding (1) Export market orientation/export performance (1) Social responsibility (1)	*BPP (36)*

By focusing on the first macro-theme (i.e., social media channels), no studies have specifically examined it from the employee perspective, thus identifying an interesting research gap. Conversely, research widely underlined the key-role of these tools from the business processes and customer perspectives. Concerning the first one, different contributions highlighted how social media support a multitude of business processes (e.g., segmentation, brand positioning, promotion, advertising, buying, after-sales), thus improving firms and marketing performance (Al-Azani and El-Alfy, 2020; Kazaishvili and Khmiadashvili, 2020; Lestari et al., 2019; Melović et al., 2020; Rebelli, 2019; Safiullin et al., 2020; Sivarajah et al., 2020; Ulas, 2019; Van Osch et al., 2019). At once, an equally relevant number of studies has also examined the social media impact from the customers’ viewpoint (Hahn, 2019; Kumar-Singh and Thirumoorthi, 2019; Rebelli, 2019; Yusmarni et al., 2020) by identifying the main advantages for them, such as their involvement and engagement in the value creation process and the access to personalized assistance services (Kazaishvili and Khmiadashvili, 2020; Sivarajah et al., 2020).

Big Data represent the second macro-theme extracted from the thematic literature review. These have been especially analysed from the business processes perspective, recognizing them as one of the most significant challenges and innovations of recent years within the DT framework. Almaslamani et al. (2020), for instance, explained how the Big Data adoption can lead firms to use intelligent market basket analysis, thus enhancing the relationship with customers. Similarly, the study of Miklosik and Evans (2020) analysed the impact of Big Data on the digital transformation of the marketing industry by examining the main challenges it faces from a data and information management viewpoint. At once, Sestino et al. (2020) provided interesting implications for marketers by underlining how the DT, enabled by Big Data, can positively influence many facets of business (e.g., collection of large-scale data allowing to identify emerging trends on consumer behaviour; creation of promotion campaigns with real-time data; creation of stronger bonds with consumers). By specifically focusing on the B2B market, the study of Sivarajah et al. (2020) demonstrated the Big Data capability to allow B2B firms to become profitable and remain sustainable through strategic operations and marketing-related business activities. Overall, the research offers interesting implications for all the stakeholders interested in understanding and exploiting the use of Big Data with the final aim of achieving business sustainability.

As for mobile marketing (mobile technology and smart apps), research has mainly examined it by focusing on the customer perspective. Indeed, mobile devices have deeply influenced customers’ behaviours and preferences toward online shopping (Sundaram et al., 2020) by also transforming them into an integral part of the value creation process. Meanwhile, mobile technology and smart apps have also been studied from the business processes viewpoint since they have become an excellent opportunity to analyse consumers in more meaningful manners, thus supporting the development of appropriate marketing strategies (Sundaram et al., 2020). Additionally, mobility, along with other digital technologies, is creating relevant opportunities for firms to transform themselves by impacting on their purchasing processes (Ulas, 2019) as well as on their distribution activities, since mobile apps represent omni-channel retail platforms allowing consumers to obtain products from different channels, such as e-commerce, modern markets, and traditional ones. In this way, the shopping experience streamlines and integrates itself across channels (Cahyadi, 2020). Conversely, even if the employee perspective has been less investigated, it represents an interesting field of study since the mobile technology is impacting, on a massive scale, the workplace (Attaran and Attaran, 2020). More in detail, it can raise employee engagement; increase productivity through the scheduling/automation of daily activities; enable real-time communications through different tools, such as group chats or one-to-one messaging. Moreover, the 5G advent could revolutionize the way employees work “in much the same way the Internet did in the 1980s” (Attaran and Attaran, 2020; p. 66). Notably, it can allow employees to (i) Fast download and upload files and documents; (ii) Quicker move data; (iii) Carry the office anywhere; (iv) Exploit resources such as real-time video interaction and smart conference/meetings rooms, thus maximizing the workplace productivity and efficiency, reducing travel time, and saving operational costs for remote employees; (v) Increase office collaboration; (vi) Synchronize and access to large amounts of data storage.

Another macro-theme widely analysed by the literature focused on the DT and marketing is Internet of Things, which represents one of the main megatrends related to the technological revolution (Hamidi et al., 2020). Extant research (e.g., Almeida et al., 2020; Chehri and Jeon, 2019) has particularly examined the main improvements provided by this technology in terms of business processes. Notably, Sestino et al. (2020) underlined how IoT can contribute to: (i) Design products/services based on consumers’ consumption experiences; (ii) Collect consumption data useful, for marketing managers, to identify new gaps, trends, or variables in understanding consumer behaviour; (iii) Identify consumers’ attitudes and choices on a large scale. At once, different studies (e.g., Almeida et al., 2020; Sestino et al., 2020) have also investigated the impact of IoT from the customer perspective by focusing on their ability to provide new types of services and high-quality products; as well as to improve the customer journey through more targeted promotions, announcements, and email marketing. Finally, even if the employee perspective represents the least investigated one, some authors (e.g., Almeida et al., 2020; Peter et al., 2020) identified several IoT advantages from this viewpoint, including the possibility of adopting mobile, flexible, team-oriented, and non-routine working methods, which allow the creation of digital workplaces; activating collaborative practices between all the staff’s levels; and communicating and disseminating corporate strategies, thus creating innovative workplaces.

Concerning the Artificial Intelligence (AI), it has been analysed from all the perspectives, especially the customer and business processes ones. Different studies investigated the advantages of the AI-based digital humans for customers, including the possibility to obtain better knowledge of their preferences and needs (Kumar-Singh and Thirumoorthi, 2019), to build an innovative and real-time relationship with the firms (Cherviakova and Cherviakova, 2018), to experience a completely new and interactive journey, and to receive personalized offers (Ianenko et al., 2019). From the processes perspective, AI significantly influences marketing processes and activities (Almeida et al., 2020; Ianenko et al., 2019; Sargut, 2019) through the analysis of the customers’ behaviours and the realization of more specific targeted profiles (Ianenko et al., 2019). AI also influences the distribution activities and, in particular, the automation of the ordering process of products and services (Cherviakova and Cherviakova, 2018). Moreover, by considering unexpected events, AI allows to recalculate new routes and to maintain constant contacts with clients and the logistics service providers. Literature (Cherviakova and Cherviakova, 2018) underlined the AI role in allowing the automatic placement of advertisements across channels, while Kumar-Singh and Thirumoorthi (2019) analysed the AI relevance also with respect to the buying/consumption process. Finally, it has been recognized the importance of AI with respect to both sales (Almeida et al., 2020) and after-sales processes, as it permits to better examine the customers’ opinions about products/services, and to identify their satisfaction level as well as the possible enhancements that could be applied to the firm’s offering. Concerning the employee perspective, AI–by representing a disruptive technology–has significantly influenced the labour relations model and, in particular, the knowledge sharing among employees (Almeida et al., 2020; Subramani, 2019; Ulas, 2019). Therefore, it becomes fundamental to enhance the employee training toward this digital tool, which is becoming more and more integrated into the workplace (Yigitcanlar et al., 2020).

By representing a multifaceted term, the Industry 4.0 has emerged as an additional macro-theme related to the DT-marketing binomial. Notably, research (e.g., Chehri and Jeong, 2019, Del Giorgio and Mon, 2019; Hamidi et al., 2020) has mainly investigated this topic from the customer and business processes perspectives, especially by focusing on the main principles behind it, namely 5c (i.e., Cooperation, Conversation, Co-creation, Cognitivity, Connectivity). This technology has created the basis of the digital ecosystem, thus offering the key ability, for firms and customers, to exchange data in real-time (Nosalska and Mazurek, 2019). By specifically focusing on the business processes perspective, an interesting point of view has been provided by Naglič et al. (2020), who analysed the Industry 4.0 macro-theme in combination with the export market orientation/export performance micro-theme. The authors offered a framework on how companies can enhance their export performance through the knowledge related to the Industry 4.0. Overall, their study detected how firms that invest in digital technologies, by effectively embracing DT, are better prepared to compete internationally, thus achieving better export performance.

Also the Machine Learning (ML) macro-theme has been mainly analysed from the business processes perspective. In particular, some studies have tried to identify the main ML implications on DT in marketing (Miklosik and Evans, 2020) by investigating the advantages this technology can bring from this perspective (Kazaishvili and Khmiadashvili, 2020; Miklosik and Evans, 2020; Polyakov and Gordeeva, 2020; Sargut, 2019). Literature focused its attention on the social media analysis (e.g., sentiment analysis on social media); packaging; product and purchasing decision-making; and advertising (e.g., interactive ad placement and targeting ads). Given that ML is a subset of AI, the literature focused on ML usually underlined, from the employee and customer perspectives, advantages very similar to the AI-related ones. More in detail, from the customers’ perspective, ML can offer personalized shopping experiences thanks to its ability to deeply know their preferences and interests. Conversely, from the employees’ viewpoint, literature mainly highlighted the key impact of ML on knowledge building and sharing (Subramani, 2019).

Concerning the online collaborative/support platforms/systems macro-theme, it emerges how it has been equally analysed from the employee and business processes perspectives. From the employee perspective, Azeredo et al. (2020) provided a proposal for the realization of an online business consulting plan through the adoption of an online collaborative platform called LexDoBusiness. More in detail, the research aimed to analyse the acceptability of this platform, which offers several benefits, especially for what concerns the levels of cohesion and cooperation between the actors involved in the business plan. In their study, Bhatnagar and Grosse (2019) underlined the relevance of a digitalized agile workplace since it allows to make employees more productive and satisfied. Similarly, Minculete and Minculete (2019) emphasized the key role of education and training actions aimed at providing staff members with the required skills for the new technologies and systems adoption. By specifically focusing on the business processes perspective, Bruskin et al. (2017) examined the development of support systems for decision-making in terms of marketing by specifically focusing on the analysis of the business effects from the adoption of similar systems.

As regards the virtual and augmented reality, literature has mainly examined it from the customer and business processes perspectives. For what concerns the first viewpoint, the majority of studies have investigated the consumers’ propensity to interact with this tool (Voronkova, 2018). Additional researches have focused their attention on the new opportunities deriving from adopting virtual and augmented reality for personalized online shopping experiences (Kim, 2020). From the business processes perspective, the virtual/augmented reality has been particularly examined with respect to the communication and advertising procedures. Notably, extant research underlined how firms can adopt the virtual reality technology to promote products and services in innovative and visual ways (Voronkova, 2018).

For what concerns the last identified macro-themes (i.e., websites/SEO; cloud infrastructure; chatbots; drones/smart robots; security protection systems; 3D print), results have already revealed a minor attention dedicated to them by the extant research (Table 5). By focusing on the websites/SEO topic, the customer and business processes perspectives represent the most investigated viewpoints. Existing studies have particularly analysed the websites topic with respect to the customer relationship management/customer connectivity/centricity (Ballestar et al., 2019) and customer experience/journey (García et al., 2019) micro-themes. With regard to the business processes perspective, the reviewed contributions have especially deepened the micro-themes of branding, communication policy, and business process efficiency. Specifically, Natorina (2020) underlined the need to implement effective marketing strategies within the DT scenario by specifically focusing on the search engine optimization (SEO). Overall, the author highlighted how the SEO represents an integral component of a successful marketing strategy since it increases the organic traffic and conversion by also enhancing the firms’ attractiveness in the sight of the Internet users.

Concerning the cloud infrastructure, it has been especially analysed from the customer perspective (Ulas, 2019) by investigating its impact on consumers’ preferences and behaviours. At the same time, the cloud infrastructure has also increased the human resources capabilities (Ulas, 2019) and improved the business processes. Notably, Kumar-Singh and Thirumoorthi (2019) shown that cloud-based digital infrastructures allow firms to increase agility, maximize resources, and improve services by also reducing operational costs. The authors also underlined the importance to analyse the impact of this technology from the demand side in order to examine how it can impact on customer preferences and behaviours.

As for the chatbots, these have been analysed from the business processes perspective and, to a lesser extent, from the employee one. Hence, an interesting research gap emerges with respect to the customer viewpoint. In particular, concerning the business processes perspective, Damnjanovic (2019) proposed a case study analysing the international positioning and go-to-market strategy of a chatbot solution, namely Weaver, which can be defined as an AI-based firm platform allowing to facilitate and simplify the sales processes. In the same year, the study of Sargut (2019) offered an insight related to the SMEs awareness, readiness, and capability in facing the DT challenge. Almost all the interviewed SMEs have confirmed to be interested in the DT subject and ready to implement chatbots and/or voice-operated machines in their business activities and processes.

Even if results underlined scarce attention of the recent literature on the robotics macro-theme (with the few identified contributions focused on the employee and business processes perspective), with the advent of the COVID-19 and the consequent reduction of human contacts, this topic will probably obtain, in the future, greater emphasis. Notably, robots will be increasingly adopted not only in order to substitute human resources but also to interact with customers. Indeed, robots “are expected to be progressively more autonomous, flexible, and cooperative” (Almeida et al., 2020, p. 102).

As for the last identified macro-themes (i.e., security protection systems and 3D print), while Li et al. (2020) emphasized the need to establish a new generation of security protection systems to increase the business processes efficiency, Ulas (2019) especially highlighted the key relevance of 3D printers in the process of new products development and design.

By considering the residual (but not irrelevant number of) contributions referring to the digitalization phenomenon as a broader macro-theme of analysis (i.e., digitalization phenomenon), it emerged an overall preference towards the adoption of a business processes and customer perspective. With regard to the former, two of the most investigated effects are the so-called “digital metrics” and “business process efficacy”. Indeed, the digitalization phenomenon has profoundly affected the analysis of the firms’ performance. Hence, the adoption of digital tools allows firms to precisely monitor and measure their social ROI (Return on Investment) in a totally new and disruptive way compared to the past. In particular, by measuring online reactions (e.g., customers’ views, likes, comments, shares), the digital metrics can contribute significantly to evaluating an ad campaign in real-time, thus permitting to modify it accordingly (e.g., Bughin et al., 2019). Moreover, a number of contributions focused on the business processes perspective has specifically analysed the role played by the digital tools in increasing the quality of the firms’ processes, thus elevating their levels of operational and organizational excellence (e.g., Kuimov et al., 2019). On the other hand, from the customer perspective, literature has mainly investigated the impact of the digitalization phenomenon on the customer journey (e.g., Taylor et al., 2020) and on the relationship management between firms and customers (e.g., Barann, 2018).

After the content analysis process has been concluded, Appendix 2 has been created, displaying the classification of the articles based on the following categorizations: (i) Author/s; (ii) Title; (iii) Source; (iv) Year of publication; (v) Analysed macro-theme; (vi) Analysed micro-theme with (vii) The respective analysis perspective (i.e., EP, CP, BPP).

## Implications and future research agenda

### General discussion

Both the descriptive and thematic results of this study provide interesting insights into the analysis of the DT-marketing topic, while crafting new propositions for future research agenda.

Descriptive data highlight the growing focus of the literature on the digital transformation-marketing topic over the last few years, with the majority of contributions published between 2019 and 2020. Notably, only nine publications have been found in the four-year period 2014–2017, while thirteen publications were reviewed in 2018, forty-five in 2019, and fifty in 2020. The publication sources are highly fragmented, given that ninety-three sources have published the 117 reviewed papers. The more cited contributions—besides being published between 2019 and 2020—have especially focused on the impact of the digitalization phenomenon on (i) Customer relationship management (Ballestar et al., 2019; Gil-Gomez et al., 2020; Hausberg et al., 2019; Peter et al., 2020; Sivarajah et al., 2020), (ii) Its coexistence with the human resources (Almeida et al., 2020; Gil-Gomez et al., 2020; Ulas, 2019; Yigitcanlar et al., 2020), and (iii) The improvement of the business processes’ performance (Sestino et al., 2020) by specifically focusing on market knowledge (Hausberg et al., 2019), communication (Ballestar et al., 2019), product development (Ulas, 2019), and sales activities (Almeida et al., 2020). Moreover, the majority of contributions here analysed has employed qualitative methods. Overall, these data, while suggesting an increasing interest by the scientific community towards the DT-marketing phenomenon, depict the absence of sources systematically and continuously dealing with this field of study, a dominant focus on certain issues, and the need to improve the adoption of quantitative methods in future research, both to validate previous research findings and to make them more generalizable.

Concerning the research questions guiding this study and, in particular the analysed themes (RQ1), these can be grouped on a twofold level concerning (i) The study of digital technologies employed in the field of marketing (*macro-themes)*, and (ii) The impact of such technologies on specific marketing activities (*micro-themes*). Overall, the literature analysis suggests an increasing pervasiveness of digital technologies in the marketing field. The use of such technologies, in fact, affects the consumer behaviour, as well as the way marketers work and marketing activities are managed and organized. In particular, it is worthy to note that DT involves the most operational marketing activities (e.g., Caliskan et al., 2020), such as sales (e.g., Almeida et al., 2020) and communication policies (e.g., Alassani and Göretz, 2019; Dasser, 2019), allowing a general increase in these processes’ quality. Meanwhile, DT also affects the analytic and strategic areas of marketing, improving the opportunities to reach new groups of consumers through the systematic use of digital technologies (such as Big Data) that allow a deeper segmentation of the market (e.g., Almaslamani et al., 2020). It supports the development of new branding strategies and the increasing visibility of brands, thanks to the use of online and social channels (e.g., Kazaishvili and Khmiadashvili, 2020; Melović et al., 2020). Moreover, DT impacts on companies’ innovativeness, helping the implementation of more effective and efficient innovative processes (Calle et al., 2020), and changes the overall relationships between firms and consumers by encouraging a customer-centric organizational culture (Cherviakova and Cherviakova, 2018, Graf et al., 2019) and the customer participation in the value creation process (Hughes and Vafeas, 2019). According to Dasser (2019), DT also implies a deeper change of marketing by elevating its strategic role as a catalytic accelerator in the digital business transformation journey.

These studies are driven by different perspectives of analysis (RQ2). The majority of research considered in this review employed a business process perspective by examining how digital technologies impact on specific marketing processes, such as sales and communication management. Nevertheless, by focusing on the main investigated topics, findings reveal that the existing research has been principally guided by a customer perspective, i.e. the way in which digital technologies are transforming customers’ behaviour, experience, and relationship with companies, followed by the business processes perspective concerning the investigation of potential improvements occurring in the area of marketing analysis and control. The employees’ perspective emerges as the less relevant among the others, despite it includes a critical part of the literature focused on the relationship between DT and human resources management. More in detail, as it emerged from our dataset, the employees’ perspective mainly characterized the first publications, investigating how digital technologies are enhancing (and requiring) the development of new marketing and business skills dealing with DT (Kwon and Park, 2017; Van Belleghem, 2015). Over the time, the scientific attention has been moved increasingly towards the customer and business processes’ perspectives. Most of the contributions published in 2020, indeed, dealt with the analysis of the DT phenomenon from the consumer viewpoint, specifically investigating the management of the customer-firm relationship (e.g., Gil-Gomez et al., 2020; Sivarajah et al., 2020), and from the business processes’ viewpoint, especially analysing the key relevance of the digital tools in measuring the firms’ performance in the social sphere (e.g., Al-Azani and El-Alfy, 2020; Lin et al., 2020). Probably, this growing interest of the research derives from the advent and unleashing, during 2020, of the COVID-19 health crisis that has led companies to almost completely digitize the relationship with customers due to the limitations imposed by the anti-COVID-19 decrees.

All these findings provide several contributions both theoretically and practically.

### Theoretical implications and research gaps

From a theoretical standpoint, this is the first study that offers a systematic and thematic review of the existing literature on DT and Marketing, while previous reviews, in the marketing field, have been very narrow in perspective. Hofacker et al. (2020), for example, examined the relevant literature on digital marketing and B2B relationships, while Miklosik and Evans (2020) focused on the impact of big data and machine learning on marketing activities. Our review, instead, addresses the DT-Marketing binomial from a wider and more comprehensive perspective, including all prior research dealing with DT in the marketing area. By doing so, this study outruns the scope of prior reviews that have been often limited to certain domains, and provides a comprehensive framework that offers a synergistic view of the existing literature, which allows a more inclusive vision and understanding about the phenomenon.

By doing so, this review also permits to highlight some relevant research gaps on which future studies might focus on.

From the combined overview between macro- and micro-themes, the main research gaps relate to the necessity of deepening the analysis of the impact of specific macro-themes from the employee (i.e., social media channels, big data, mobile marketing, Artificial Intelligence, Industry 4.0, Cloud infrastructure, Virtual/augmented reality, and websites), customer (i.e., Social media channels, Big Data, Industry 4.0; Internet of Things; Machine Learning; Websites; Chatbots), and business processes perspective (i.e., Mobile technology; Artificial Intelligence; Virtual/Augmented reality; Cloud infrastructure; Drones/Smart robots).

**Fig. 3:**
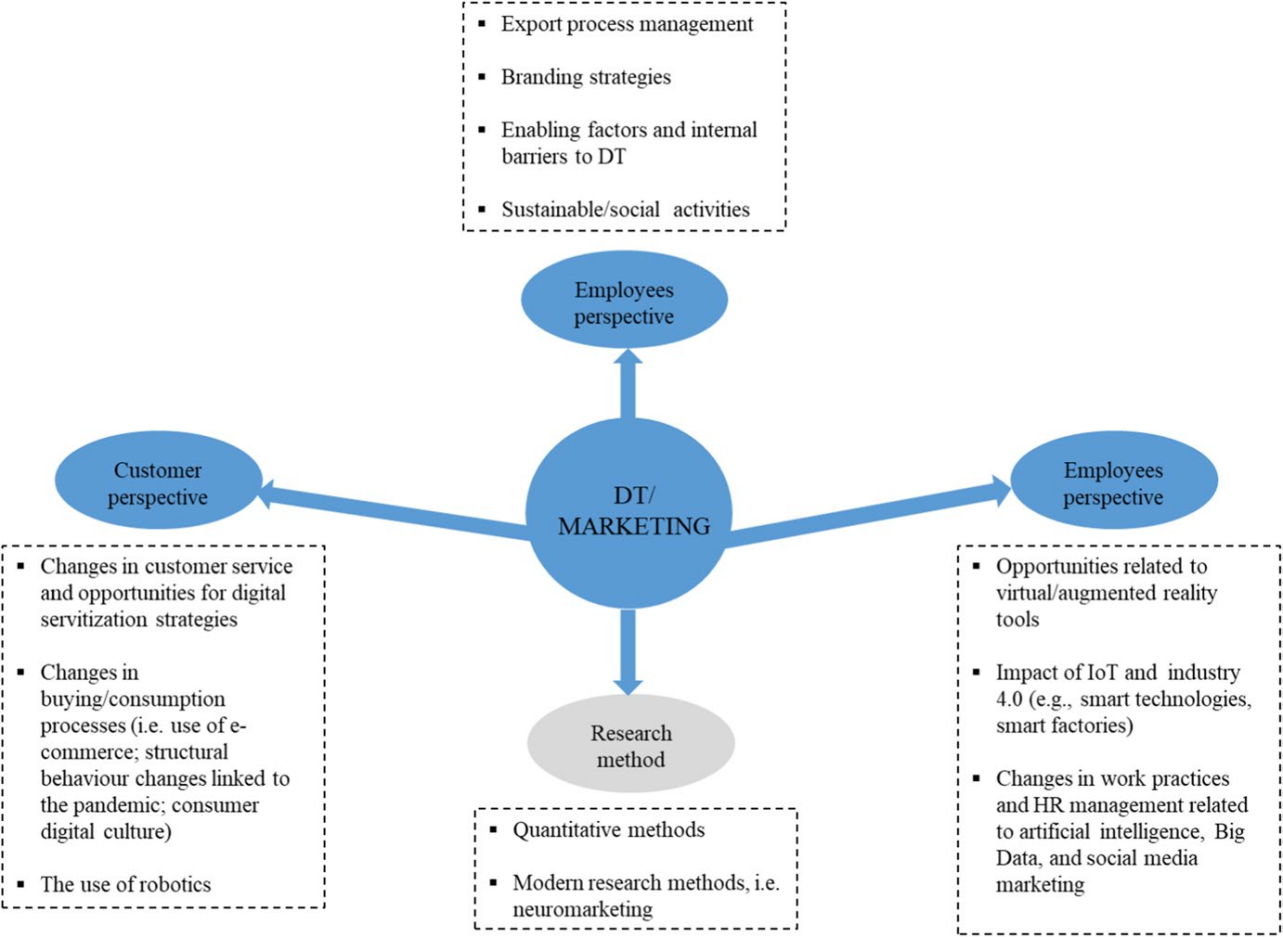
Areas of future research on DT and Marketing

Besides that, the variety of analysed studies, while manifesting the pervasive use of digital technologies in the marketing field, reveals that the extant literature is quite fragmented and even sparse with regard to specific micro-themes. Some topics, like customer service, smart factories, consumer behaviour, have been investigated by few contributions, thus highlighting potential opportunities for further studies. In this respect, our review can be viewed as a solid basis for additional discussion and research within each perspective emerged from the analysis (see Fig. 3).

More in detail, the findings reveal that the employees’ perspective is worthy of further attention, as it is the less investigated one. Although several contributions (n. 21) focused on DT and human resources by highlighting the need for enhanced skills in using technology (e.g., Dethine et al., 2020; Ulas, 2019), the development of new prominent job positions for the future (e.g. digital marketing manager; social media manager; big data/data analyst) (e.g., Di Gregorio et al., 2019, Hafezieh and Pollock, 2018), and the critical role of training and educational actions enhancing the appropriate use of digital technologies in the marketing context (Yigitcanlar et al., 2020), other themes have been under-investigated. In particular, only two papers dealt with the subject of smart technologies by investigating how they can help cities to face the increasing urbanization (Visan and Ciurea, 2020), and their importance for establishing a predictive maintenance of production systems, which can increase the process quality (Chehri and Jeon, 2019). The application of smart technologies can also redefine the way people conduct business, bringing benefits in terms of productivity and employee well-being (Papagiannidis and Marikyan, 2020). Thus, there is scope for considering, in future research, how smart technologies are used to conduct marketing activities and how they are changing the way marketers work and organize their processes.

Under the customer perspective, several topics might deserve attention in future research. Most of the analysed contributions addressed the impact of DT on firms/customers relationships, highlighting the need for new forms of interaction and collaborations with customers due to changes in behaviour. Several scholars recognized the advantage of DT as it allows to establish innovative and real-time relationships with the market (e.g. Almaslamani et al., 2020), to engage customers in the value creation process (e.g. Saravanabhavan et al., 2020; Taylor et al., 2020), and to provide customers with more interactive and personalized experiences (e.g. Taylor et al., 2020; Venermo et al., 2020). However, our findings suggest that other topics, although relevant, are still at the begin of their investigation. Only three contributions focused on customer service (Lieberman, 2019; Lin et al., 2020; Safiullin et al., 2020), especially revealing the role of digital tools in the online customer service and the importance of electronic services for improving customer satisfaction (Lin et al., 2020). A recent study (Galvani and Bocconcelli, 2021) revealed that a new business model is emerging in the BtoB context characterized by an overall revolution towards the digital servitization strategy, which replaces the traditional product-centric paradigm. Hence, future research could investigate whether and how the digital servitization strategy is currently implemented in the BtoC context, which opportunities and benefits can offer—especially concerning the firm-customers’ relationship, and how marketing managers can act to face the imperative complexity linked to its adoption. Another theme receiving increasing—but still few—attention concerns the buying/consumption processes. Few scholars analysed the impact of digital tools on customers buying processes (Kim, 2020), the increasing use of e-commerce (Cahyadi, 2020), and structural changes occurring in consumption during COVID-19 pandemic (Kim, 2020). However, the identification of consumption patterns and trends has been always a central topic in the marketing literature, as proved by the wide number of literature reviews, even focused on specific areas such as electronic word of mouth (Huete-Alcocer, 2017), online consumption (Hwang and Jeong, 2016), or COVID-19 crisis (Cruz-Cárdenas et al., 2021). Therefore, continuing the research on DT and consumption/buying behaviour is desirable to properly adapt the marketing management with the aim of satisfying specific market needs and expectations, as well as realizing a stronger engagement of customers in the value creation process, which is getting more and more attention within the recent marketing and management literature (Fan and Luo, 2020). Besides, future studies on DT and consumption/buying behaviour might also employ modern research methods, such as neuromarketing. We found only one contribution based on the analysis of the use of advanced methods in the field of artificial neural networks (Polyakov and Gordeeva, 2020). However, neuromarketing could contribute to overcome several limitations associated with traditional data collection method (i.e. self-report data), while allowing to capture unconscious brain processes that relate to consumer decision-making (Sung et al., 2021).

Finally, an additional space for future research emerged from our review of publications is related to the business processes perspective. This area shows the greatest potential for exploration, given the richness of themes it includes. In this perspective, in fact, except for some activities related to marketing analysis and control, and operational policies—especially product and communication ones—the rest of the literature appears very fragmented and scarce. Notably, specific attention might be devoted to DT and export process management, as Naglič et al. (2020) found that firms which invest in DT are better prepared to compete internationally and achieve better export performance; branding strategies, as they have been recognized as critical for marketing competitiveness (Kazaishvili and Khmiadashvili, 2020), drivers/barriers and risks associated to DT implementation in the marketing areas; and sustainable/social opportunities and treats that digital technologies can bring with them, as they can differently affect the success of human-centric marketing programs in the digital environment (Agafonova et al., 2020). All these topics have been very little investigated by previous research, while deserving increasing attention given their relation with companies’ success and long-term competitiveness.

### Practical implications

Regarding the practical contributions, our review offers a number of suggestions to marketing managers as it analyses the DT-Marketing binomial both internally (i.e. on the firm level) and externally (i.e. on the inter-firm level). This approach results from the recognition of different perspectives of analysis adopted by prior research, which combines contributions focused on the management of internal processes and marketing activities with studies investigating the DT phenomenon from a customer-based viewpoint. Consistent with our twofold approach of analysis, the practical implications deserving particular attention can be summarized into two main groups concerning (i) The changing role of marketing in the company resulting from the increasing use of digital technologies, and (ii) The changing relationships between firms (and marketing) and external stakeholders (especially consumers).

Literature suggests that DT could improve the strategic role of marketing within the firm, as it enhances the marketing capability to analyse the market scenario and to develop a more comprehensive understanding of the demand (Papagiannopoulos and Lopez, 2018), which, in turn, can support new products development that are better aligned with customers’ expectations (Kuimov et al., 2019). Overall, digital technologies can help companies to become data-driven subjects, where marketing covers a central position given its informative and intra-firm coordinating role. However, the full exploitation of such opportunities means change, at both cultural and structural levels. Our review, in particular, reveals that DT requires a cultural upgrading, to cope with DT and its effects on the business (e.g., Álvarez-Flores et al., 2018; Dethine et al., 2020), the enhancement of internal competences in the field of technology (Ulas, 2019), the development of new job positions (Di Gregorio et al., 2019), and the gradual adoption of new working habits and patterns (Minculete and Minculete, 2019). Of course, educational and training activities become prominent to support such changes, passing through the acquisition of new skills from the market labour, as well as through the enhancement and conversion of internal resources. Besides training programs, organized both internally and externally in collaboration with private and public institutions such as high schools and universities, companies could also provide ad hoc rewards to encourage the commitment and interest of marketing employees in digital innovation.

The second group of advices concerns the changing relationships between firms (and marketing) and external stakeholders (especially consumers). DT affects the customer behaviour and changes his ability to communicate with the company (e.g., Caliskan et al., 2020), to be engaged in the value creation process (e.g., Taylor et al., 2020), and to live personalized consumption experiences (e.g., Fokina and Barinov, 2019). All this implies a general re-thinking about the firm-customer relationship management. Consumers are becoming empowered subjects that no longer accept the role of passive receivers of marketing initiatives (Acar and Puntoni, 2016) and companies need to open to their customers, accepting their participation in the marketing decision-processes. Undoubtedly, the use of social-media platforms can be decisive to create engaging content and connect with customers, improving the interaction and the dialog with them, for example by responding to a specific comment or complaint (Acar and Puntoni, 2016). However, digital technologies can be also used to create more advanced tools that are able to strengthen the connection between brands and customers, such as crowdsourcing, co-creation, and/or brand communities. These platforms can be used successfully by firms to improve the dialog with customers and their involvement in several marketing processes, such as the selection of an advertising campaign and/or the creation of new product ideas.

## Conclusions and limitations

This study provides a synergistic view of existing literature on the binomial DT-Marketing by detecting the main themes investigated and the relative approach of analysis characterizing prior research. It offers a comprehensive framework, which combines both internal and external perspectives to analyse the impact of DT on all the activities on which the traditional marketing management is based, dealing with the areas of market information and knowledge, marketing strategies, and operational policies. Moreover, it also considers how the binomial DT-Marketing has been investigated in the wider context of the firm, by taking into account the organizational, human, and structural changes associated to the adoption of digital tools in the marketing field. By doing so, our review synthetizes prior research on DT and marketing, provides suggestions for future research directions, and offers practical implications for marketing managers. Notwithstanding these contributions, the current study presents its limitations.

First, despite the adoption of a rigorous methodological approach in conducting the review, some pertinent studies are likely to have been omitted, as the research was based on a certain combination of keywords in the search string and, above all, it was focused on a single database, i.e. Scopus. While Scopus covers all top journals and scientific publications, containing most references from other databases (Guerrero et al., 2015), it could not necessarily capture all contributions due to retrieval conditions and data source limitations. Therefore, a future updating of the current review should consider other leading databases, such as ISI, Web of Science, and EBSCO, which are also appreciated for their comprehensiveness in the field of peer-review literature and management research (Schryen, 2015).

Second, the analysis was focused on recent literature published between 2014 and 2020. Although the literature on DT has gained in interests over the last years, especially from 2014 (Vaska et al., 2021), there may be articles and authors that do not come under our review. Moreover, the results are valid only for the specific time-period we considered in this study. Therefore, a future review (e.g., including the years before 2014 and the years after 2020) might extend the time framing as new research works could modify our findings, particularly in light of the constant development of digital technologies and marketing scenarios.

Finally, in our review, the qualitative analysis and descriptions are based on the research team interpretation of the selected references, which is subject to the limitations of human judgments. While it is impossible to eliminate human interpretation in scientific research, as it is critical to make the results more meaningful (Zhu et al., 2021), a future review could combine bibliometric analysis methods (e.g., citation, main path analysis) with subjective analysis, to reduce the influence of human interpretation and provide a more accurate description of DT-Marketing research. To this end, the use of appropriate software tools, such as VOSviewer, should provide notable improvements to the research quality, as it allows to create large bibliometric maps, which offer a clear and easy way to manage the visualization of data analysis (Shah et al., 2020).

## Appendix 1

See Table

**Table 10:** Macro-themes and main definitions

Macro-themes	Definitions extracted from the reviewed contributions focused on the marketing domain
Social media channels/Social media marketing	[1] Social media “are valuable forums through which customers can directly contact brands to exchange experiences” (*Melović *et al *., p. 4*); [2] “Social media marketing is used across sectors and refers to “the utilization of social media technologies, channels, and software to create, communicate, deliver, and exchange offerings that have value for an organization’s stakeholders” (*Melović *et al *., p. 4);* [3] “Social media is a communications and networking tool whose popularity has been constantly rising since its users can connect, share, and interact among themselves” (*Melović *et al*., pp. 4–5);* Social media can be defined as “a pertinent platform for public engagement, inter-organizational relationships, and public information” (Sivarajah et al., 2020*, p. 165*); [4] Social media can be defined as “a group of Internet-based applications that build on the ideological and technological foundations of Web 2.0” (Kaczorowska-Spychalska, 2018*, p. 15*)
Big Data	[1] Structured data such as organizational databases, and unstructured data generated by new communication technologies, as well as images, videos, audio (Sestino et al., 2020*, p. 1*); [2] Big Data is the term that can be used to refer to this extremely broad set of data, which, therefore, needs special tools to store, extract, organize, and transform the data into information that can be analyzed widely and in a short time (Almeida et al., 2020*, p. 100*); [3] Big Data is an evolving term that is used to describe any large amount of structured, semi-structured or unstructured data that has a potential to be mined for information (Ulas, 2019*, p. 665*)
Mobile marketing (Mobile technology; Smart apps)	[1] Digital mobile application can be a new business model in selling products from companies (Cahyadi, 2020*, p. 3*); [2] Mobile marketing “can be defined as the planning and execution of all mobile-based marketing activities that influence a shopper, from the initial shopping trigger, to the purchase, consumption and recommendation stages” (Kaczorowska-Spychalska, 2018, p. 15)
Internet of Things	[1] “The IoT can be seen as the network of physical objects that contains technologies and software that enables them to communicate and interact intelligently internally or with their external environment over the Internet” (Almeida et al., 2020*, p. 100*); [2] “Whereas the term “Internet” refers to a virtual network-oriented vision of technology, the term “Things” emphasizes the objects that can be integrated into a technological framework” (Sestino et al., 2020*, p. 2*); [3] “IoT is meant as an information infrastructure around the world where unique physical and virtual images have been identified and linked to the internet which have sparked innovative, sophisticated services, and created a simpler and smarter life” (Hamidi et al., 2020, *p. 1540*); [4] “Internet of things is a technology based on data transfer between devices over internet. It consists of interconnected devices from simple sensors to smartphones and wearable devices” (Ulas, 2019*, p. 664*)
Artificial Intelligence	[1] “AI can be defined as machines or computers that mimic cognitive functions that humans associate with the human mind, such as learning and problem solving. AI is a branch of computer science that perceives its environment and acts to maximize its chances of success” (Yigitcanlar et al., 2020*, p. 3*); [2] “Artificial intelligence investigates how a human brain thinks and how people learn and decide as they try to solve a problem, and it imitates the results of this study with smart software. Artificial intelligence does not act upon programmer’s mind, it learns, understands and judges itself” (Ulas, 2019*, p. 664*)
Industry 4.0 and 5C	[1] “The main difference of Industry 4.0 from the other industrial revolutions is to connect people, machines, and objects to improve the efficiency of production while involving the customers to all processes” (Caliskan et al., 2020, *p. 1252*); [2] “It concerns a shift in the production practice—from mass to personalised production—which results in greater flexibility of production processes and provides means to satisfy the individual needs of different customers more effectively” (Nosalska and Mazurek, 2019, *p. 10*); [3] The main principles of marketing for the Fourth Industrial Revolution are: Cooperation, Conversation, co-creation, cognitivity, connectivity” (Nosalska and Mazurek, 2019); [4] “Industry 4.0 involved digital transformation that describe the future of industry” (Hamidi et al., 2020, *p. 1540*); [5] “In fourth industrial revolution, it is foreseen that manufacturing process is digitized, machines are directly connected to each other and personalized manufacturing is possible, besides that, environment is less polluted as a result of productivity growth, avoiding of excessive use of energy and water sources. As manufacturing becomes flexible with digital factories, manufacturing meeting less and personal product demand becomes possible” (Ulas, 2019*, p. 664*)
Machine Learning	[1] Machine learning (ML) is strictly connected to the computer science domain. It allows personal computers to become more efficient, in a specific task, through experience. ML can be applied, in the marketing domain, in the social media analysis, product and purchasing decision-making, and advertising (Miklosik and Evans, 2020)
Online collaborative/ support platforms/systems	[1] Online collaborative systems are platforms providing several benefits, especially for what concerns the levels of cohesion and cooperation between the actors involved (Azeredo et al., 2020)
Virtual/ Augmented Reality	[1] “Augmented reality (AR) is described as the extension of physical reality by adding layers of computer generated information to the real environment. Information in this context could be any kind of virtual object or content, including text, graphics, video, sound, haptic feedback” (Ulas, 2019*, p. 665*)
Websites/SEO	[1] “Digital transformation and the high level of offline and online competition have a significant impact on business, which emphasizes the relevance and need to implement the successful relevant marketing strategy that includes the search engine optimization (SEO). The SEO increases the business visibility on the Internet in comparison with priority competitors, promotes better communication and interaction in accordance with varied requests of online buyers (customers, clients)” (Natorina, 2020*, p. 83*);
Cloud Infrastructure	“It is a general term of internet based information services providing computer sources which are used or shared between users on request for computers and other devices” (Ulas, 2019, p. 665)
Chatbot’s	[1] “Chatbots are an innovative way of interacting with customers and they are well-known for their advantages such as: availability, handling capacity, cost efficiency and personalization. They are present wherever the customers are, responding to requests immediately. A chatbot is an instant messaging account that able to provide services using instant messaging frameworks with the aim of providing conversational services to users in an efficient manner” (Damnjanovic, 2019*, p. 40*); [2] “They are software applications which are designated with the intent of backing up users in service sectors and imitate written or verbal human speaking” (Ulas, 2019*, p. 665*)
Drones/Smart Robots	“Vehicles with reduced or no human intervention. Robotics shift the labour/capital mix while managing societal expectations” (Ulas, 2019, p. 666)
Security Protection systems	The security protection systems concern the following boundary types: Information Intranet and Third Party Boundary; Information internal and external network boundary; Security Boundary between Horizontal Domains of Information Intranet, and Vertical Security Boundary of Information Intranet. The main security protection technologies are: Hardware Firewall or Software Firewall; Virtual Firewall Technology; Access Control Technology between VLANs (Virtual Local Area Network) (Li et al., 2020)
3D Print	“It is a device quickly producing models which are designed in computer or prepared in 3D by using various materials without any mould or fixture” (Ulas, 2019, p. 665)

10.

## Appendix 2

See Table

**Table 11:** List of reviewed articles

Author/s	Title	Source	Year	Macro trends	Micro trends	Analyzed perspective
Almeida, F., Duarte Santos, J., Augusto Monteiro, J	The Challenges and Opportunities in the Digitalization of Companies in a Post-COVID-19 World	IEEE Engineering Management Review	2020	AI; IoT; Big Data; Robotics	Human resources	EP
Andriole S. J	The hard truth about soft digital transformation	IT Professional	2020	Digitalization phenomenon		
Attaran, M., Attaran, S	Digital Transformation and Economic Contributions of 5G Networks	International Journal of Enterprise Information Systems	2020	Mobile technology/smart apps		
Azeredo, H., Reis, J.L., Pinto, A.S	The LexDoBusiness Collaborative Platform	Smart Innovation, Systems and Technologies	2020	Online collaborative platforms		
Calle, A., Freije, I., Ugarte, J.V., Larrinaga, M.Á	Measuring the impact of digital capabilities on product-service innovation in Spanish industries	International Journal of Business Environmen	2020	Digitalization phenomenon		
Dethine, B., Enjolras, M., Monticolo, D	Digitalization and SMEs’ export management: Impacts on resources and capabilities	Technology Innovation Management Review	2020	Digitalization phenomenon		
Peter, M.K., Kraft, C., Lindeque, J	Strategic action fields of digital transformation: An exploration of the strategic action fields of Swiss SMEs and large enterprises	Journal of Strategy and Management	2020	IoT		
Rahimian, O	Managing your digital transformation	Proceedings of the Annual Offshore Technology Conference	2020	Digitalization phenomenon		
Shkarlet, S., Dubyna, M., Shtyrkhun, K., Verbivska, L	Transformation of the paradigm of the economic entities development in digital economy	WSEAS Transactions on Environment and Development	2020	Digitalization phenomenon		
Bhatnagar, S., Grosse, M	Future workplace organisation: How digitisation affects employees' job satisfaction in agile workplaces	International Journal of Product Development	2019	Online collaborative platforms		
Dasser, M	Marketing, the change catalyst for digital business transformation: Lessons learned from the modernisation of a B2B marketing organisation	Journal of Brand Strategy	2019	Digitalization phenomenon		
Di Gregorio, A., Maggioni, I., Mauri, C., Mazzucchelli, A	Employability skills for future marketing professionals	European Management Journal	2019	Digitalization phenomenon		
Fiodorov, I., Ochara, N.M	The impact of digital transformation on economic of BRICS countries	CEUR Workshop Proceedings	2019	Digitalization phenomenon		
Minculete, G., Minculete, S	Approaches to companies’ personnel educationand training in the field of digital marketing	eLearning and Software for Education Conference	2019	Online collaborative platforms		
Subramani, S	Transforming the enterprise with applied artificial intelligence	Applied Marketing Analytics	2019	AI; Machine Learning		
Ulas, D	Digital Transformation Process and SMEs	Procedia Computer Science	2019	Mobile technology/smart apps; Cloud infrastructure; 3D print; Robotics; Big Data; AI; IoT; Chatbots; Industry 4.0; Virtual/Augmented reality		
Zimand Sheiner, D., Earon, A	Disruptions of account planning in the digital age	Marketing Intelligence and Planning	2019	Big Data		
Álvarez-Flores, E.P., Núñez-Gómez, P., Olivares-Santamarina, J.P	Professional profiles and work market access for graduates in Advertising and Public relations: From specialization to hybridization	Profesional de la Informacion	2018	Digitalization phenomenon		
Hafezieh, N., Pollock, N	The rise of new expertise in digital technologies: The 'doing' of expert knowledge and the role of the organisation	International Conference on Information Systems 2018	2018	Digitalization phenomenon		
Kwon, E.H., Park, M.J	Critical factors on firm’s digital transformation capacity: Empirical evidence from Korea	International Journal of Applied Engineering Research	2017	Digitalization phenomenon		
Van Belleghem, S	When digital becomes human	Journal of Direct, Data and Digital Marketing Practice	2015	Digitalization phenomenon		
Almeida, F., Duarte Santos, J., Augusto Monteiro, J	The Challenges and Opportunities in the Digitalization of Companies in a Post-COVID-19 World	IEEE Engineering Management Review	2020	AI; IoT; Big Data; Robotics	MarTech	EP
Andriole S. J	The hard truth about soft digital transformation	IT Professional	2020	Digitalization phenomenon		
Caliskan, A., Özkan Özen, Y.D., Ozturkoglu, Y	Digital transformation of traditional marketing business model in new industry era	Journal of Enterprise Information Management	2020	Industry 4.0		
Calle, A., Freije, I., Ugarte, J.V., Larrinaga, M.Á	Measuring the impact of digital capabilities on product-service innovation in Spanish industries	International Journal of Business Environmen	2020	Digitalization phenomenon		
Federico, F	A journey of digital marketing transformation: From distributed solo players to embedded digital excellence	Journal of Digital and Social Media Marketing	2020	Digitalization phenomenon		
Krasonikolakis, I., Tsarbopoulos, M., Eng, T.-Y	Are incumbent banks bygones in the face of digital transformation?	Journal of General Management	2020	Digitalization phenomenon		
Rahimian, O	Managing your digital transformation	Proceedings of the Annual Offshore Technology Conference	2020	Digitalization phenomenon		
Shkarlet, S., Dubyna, M., Shtyrkhun, K., Verbivska, L	Transformation of the paradigm of the economic entities development in digital economy	WSEAS Transactions on Environment and Development	2020	Digitalization phenomenon		
Yigitcanlar, T., Kankanamge, N., Regona, M., Maldonado, A.R., Rowan, B., Ryu, A., Desouza, K.C., Corchado, J.M., Mehmood, R., Li, R.Y.M	Artificial intelligence technologies and related urban planning and development concepts: How are they perceived and utilized in Australia?	Journal of Open Innovation: Technology, Market, and Complexity	2020	AI		
Chantayarkul, A., Ayuthaya, S.D.N., Kiattisin, S	The Marketing Strategy for Enhancing the Competitiveness of Local Traditional Stores in Thailand	59th Annual Conference of the Society of Instrument and Control Engineers of Japan	2019	Digitalization phenomenon		
Lekunze, N.J., Luvhengo, U	Framework for agriculture fresh produce market hub in a rural area: Application of bricks and click method in Taung, South Africa	Asia Life Sciences	2019	Mobile technology/smart apps		
Barann, B	An is-perspective on omni-channel management: Development of a conceptual framework to determine the impacts of touchpoint digitalization on retail business processes	26th European Conference on Information Systems	2018	Digitalization phenomenon		
Hafezieh, N., Pollock, N	The rise of new expertise in digital technologies: The 'doing' of expert knowledge and the role of the organisation	International Conference on Information Systems 2018	2018	Digitalization phenomenon		
Kwon, E.H., Park, M.J	Critical factors on firm’s digital transformation capacity: Empirical evidence from Korea	International Journal of Applied Engineering Research	2017	Digitalization phenomenon		
Van Belleghem, S	When digital becomes human	Journal of Direct, Data and Digital Marketing Practice	2015	Digitalization phenomenon		
Visan, M., Ciurea, C	Smart City: Concepts and two Relevant Components	International Journal of Computers, Communications and Control	2020	Big Data; Cloud; Mobile Technology/smart apps	Smart Factories	EP
Chehri, A., Jeon, G	The Industrial Internet of Things: Examining How the IIoT Will Improve the Predictive Maintenance	Smart Innovation, Systems and Technologies	2019	Industry 4.0; IoT		
Almaslamani, F., Abuhussein, R., Saleet, H., AbuHilal, L., Santarisi, N	Using big data analytics to design an intelligent market basket-case study at sameh mall	International Journal of Engineering Research and Technology	2020	Big Data	Customer relationship management/customer connectivity/centricity	CP
Cahyadi, I	Developing Digital Application to Improve Business Process Sustainability in An Indonesian Fast Moving Consumer Goods Company	Journal of Physics: Conference Series	2020	Mobile technology/smart apps		
Caliskan, A., Özkan Özen, Y.D., Ozturkoglu, Y	Digital transformation of traditional marketing business model in new industry era	Journal of Enterprise Information Management	2020	Industry 4.0		
Gil-Gomez, H., Guerola-Navarro, V., Oltra-Badenes, R., Lozano-Quilis, J.A	Customer relationship management: digital transformation and sustainable business model innovation	Economic Research-Ekonomska Istrazivanja	2020	Digitalization phenomenon		
Peter, M.K., Kraft, C., Lindeque, J	Strategic action fields of digital transformation: An exploration of the strategic action fields of Swiss SMEs and large enterprises	Journal of Strategy and Management	2020	IoT		
Rolbina, E.S., Novikova, E.N., Sharafutdinova, N.S., Martynova, O.V., Akhmetshin, R.M	Analysis and assessment of quality of medical services in conditions of digital transformation	Advances in Intelligent Systems and Computing	2020	Digitalization phenomenon		
Shkarlet, S., Dubyna, M., Shtyrkhun, K., Verbivska, L	Transformation of the paradigm of the economic entities development in digital economy	WSEAS Transactions on Environment and Development	2020	Digitalization phenomenon		
Sivarajah, U., Irani, Z., Gupta, S., Mahroof, K	Role of big data and social media analytics for business to business sustainability: A participatory web context	Industrial Marketing Management	2020	Big data; social media channels		
Taylor, S.A., Hunter, G.L., Zadeh, A.H., Delpechitre, D., Lim, J.H	Value propositions in a digitally transformed world	Industrial Marketing Management	2020	Digitalization phenomenon		
Ballestar, M.T., Grau-Carles, P., Sainz, J	Predicting customer quality in e-commerce social networks: a machine learning approach	Review of Managerial Science	2019	Websites/SEO; Machine learning		
García, J.J.L, Lizcano, D., Ramos, C.M.Q., Matos, N	Digital marketing actions that achieve a better attraction and loyalty of users: An analytical study	Future Internet	2019	Websites/SEO		
Graf, M., Peter, M., Gatziu-Grivas, S	Foster strategic orientation in the digital age: A methodic approach for guiding SME to a digital transformation	Lecture Notes in Business Information Processing	2019	Digitalization phenomenon		
Hahn, S.M.L	Influence of Digital Transformation on the Customer Relationship	Advances in Intelligent Systems and Computing	2019	Mobile technology/smart apps; social media channels		
Handfield, R	Shifts in buyer–seller relationships: A retrospective on Handfield and Bechtel	Industrial Marketing Management	2019	Digitalization phenomenon		
Hausberg, J.P., Liere-Netheler, K., Packmohr, S., Pakura, S., Vogelsang, K	Research streams on digital transformation from a holistic business perspective: a systematic literature review and citation network analysis	Journal of Business Economics	2019	Virtual/Augmented Reality		
Kumar-Singh, A., and Thirumoorthi P	The impact of digital disruption technologies on customer preferences: The case of retail commerce	International Journal of Recent Technology and Engineering	2019	Social media channels; Mobile technology/smart apps; AI; Big Data; Cloud; Virtual/Augmented reality; IoT		
Ianenko, M., Ianenko, M., Huhlaev, D., Martynenko, O	Digital transformation of trade: Problems and prospects of marketing activities	IOP Conference Series: Materials Science and Engineering	2019	AI		
Nosalska, K., Mazurek, G	Marketing principles for Industry 4.0—a conceptual framework	Engineering Management in Production and Services	2019	Industry 4.0		
Subramani, S	Transforming the enterprise with applied artificial intelligence	Applied Marketing Analytics	2019	AI; Machine Learning		
Barann, B	An is-perspective on omni-channel management: Development of a conceptual framework to determine the impacts of touchpoint digitalization on retail business processes	26th European Conference on Information Systems	2018	Digitalization phenomenon		
Papagiannopoulos, N., Lopez, J.F.G	Understanding and predicting passenger behaviours through data analytics	Journal of Airport Management	2018	Big Data		
Cherviakova, V., Cherviakova, T	Value opportunities for automotive manufacturers in conditions of digital transformation of the automotive industry	Journal of Applied Economic Sciences	2018	AI; Mobile technology/smart apps		
Serrano-Cobos, J	Internet technology trends: Towards a paradigm shift	Profesional de la Informacion	2016	AI; Big Data; IoT		
Van Belleghem, S	When digital becomes human	Journal of Direct, Data and Digital Marketing Practice	2015	Digitalization phenomenon		
Hamidi, S.R., Muhamad Yusof, M.A., Shuhidan, S.M., Kadir, S.A	Ir4.0: Unmanned store apps	Indonesian Journal of Electrical Engineering and Computer Science	2020	IoT; Mobile technology/smart apps; Industry 4.0	Customer experience/Journey	CP
Taylor, S.A., Hunter, G.L., Zadeh, A.H., Delpechitre, D., Lim, J.H	Value propositions in a digitally transformed world	Industrial Marketing Management	2020	Digitalization phenomenon		
Venermo, A., Rantala, J., Holopainen, T	From Sales Funnel to Customer Journey	Advances in Intelligent Systems and Computing	2020	Digitalization phenomenon		
Dasser, M	Marketing, the change catalyst for digital business transformation: Lessons learned from the modernisation of a B2B marketing organisation	Journal of Brand Strategy	2019	Digitalization phenomenon		
Fokina, O., Barinov, S	Marketing concepts of customer experience in digital economy	E3S Web of Conferences	2019	Digitalization phenomenon		
García, J.J.L, Lizcano, D., Ramos, C.M.Q., Matos, N	Digital marketing actions that achieve a better attraction and loyalty of users: An analytical study	Future Internet	2019	Websites/SEO		
Kuimov, V.V., Yushkova, L.V., Scherbenko, E.V., Gunyakov, Y.V	Digital Transformations in the Development of Cooperative Network Interactions	ACM International Conference Proceeding Series	2019	Digitalization phenomenon		
Kumar-Singh, A., and Thirumoorthi P	The impact of digital disruption technologies on customer preferences: The case of retail commerce	International Journal of Recent Technology and Engineering	2019	Social media channels; Mobile technology/smart apps; AI; Big Data; Cloud; Virtual/Augmented reality; IoT		
Lieberman, M	How ‘the new customer buyer’s journey’ is reshaping the way you strategically manage your brand	Journal of Brand Strategy	2019	Digitalization phenomenon		
Nosalska, K., Mazurek, G	Marketing principles for Industry 4.0—a conceptual framework	Engineering Management in Production and Services	2019	Industry 4.0		
Saito, T., Nishio, K	Approach to building of data utilization platform to realize optimization of customer experience	Fujitsu Scientific and Technical Journal	2019	Digitalization phenomenon		
Hafezieh, N., Pollock, N	The rise of new expertise in digital technologies: The 'doing' of expert knowledge and the role of the organisation	International Conference on Information Systems 2018	2018	Digitalization phenomenon		
Heuchert, M., Barann, B., Cordes, A.-K., Becker, J	An IS perspective on omni-channel management along the customer journey: Development of an entity-relationship-model and a linkage concept	MKWI 2018	2018	Digitalization phenomenon		
Papagiannopoulos, N., Lopez, J.F.G	Understanding and predicting passenger behaviours through data analytics	Journal of Airport Management	2018	Big Data		
Persson, M., Grundstrom, C., Väyrynen, K	A case for participatory practices in the digital transformation of insurance	31st Bled eConference: Digital Transformation:	2018	Digitalization phenomenon		
Escadafal, A	From territories to tourist areas: Ending some illusion?	Sud-Ouest Europeen	2015	Digitalization phenomenon		
Van Belleghem, S	When digital becomes human	Journal of Direct, Data and Digital Marketing Practice	2015	Digitalization phenomenon		
Almaslamani, F., Abuhussein, R., Saleet, H., AbuHilal, L., Santarisi, N	Using big data analytics to design an intelligent market basket-case study at sameh mall	International Journal of Engineering Research and Technology	2020	Big Data	Customer behavior	CP
Rados, I., Hajnic, M., Rados, I	Digital transformation of monitoring customer behaviour in the cars sales	MIPRO 2020—Proceedings	2020	Digitalization phenomenon		
Del Giorgio, H.R., Mon, A	Usability in ICTs for industry 4.0	Communications in Computer and Information Science	2019	Industry 4.0		
Fokina, O., Barinov, S	Marketing concepts of customer experience in digital economy	E3S Web of Conferences	2019	Digitalization phenomenon		
Hahn, S.M.L	Influence of Digital Transformation on the Customer Relationship	Advances in Intelligent Systems and Computing	2019	Mobile technology/smart apps; social media channels		
Kumar-Singh, A., and Thirumoorthi P	The impact of digital disruption technologies on customer preferences: The case of retail commerce	International Journal of Recent Technology and Engineering	2019	Social media channels; Mobile technology/smart apps; AI; Big Data; Cloud; Virtual/Augmented reality; IoT		
Ianenko, M., Ianenko, M., Huhlaev, D., Martynenko, O	Digital transformation of trade: Problems and prospects of marketing activities	IOP Conference Series: Materials Science and Engineering	2019	AI		
Lieberman, M	How ‘the new customer buyer’s journey’ is reshaping the way you strategically manage your brand	Journal of Brand Strategy	2019	Digitalization phenomenon		
Rebelli, H	Study on effect of social media on retail buying behaviour	International Journal of Advanced Science and Technology	2019	Social media channels		
Kaczorowska-Spychalska, D	Shaping consumer behaviour in the fashion industry by interactive communication forms	Fibres and Textiles in Eastern Europe	2018	Social media channels; Mobile technology/smart apps		
Papagiannopoulos, N., Lopez, J.F.G	Understanding and predicting passenger behaviours through data analytics	Journal of Airport Management	2018	Big Data		
Saravanabhavan, H., Raman, S., Maddulety, K	Value Creation from the Impact of Business Analytics	IFIP Advances in Information and Communication Technology	2020	Digitalization phenomenon	Value co-creation/value proposition	CP
Taylor, S.A., Hunter, G.L., Zadeh, A.H., Delpechitre, D., Lim, J.H	Value propositions in a digitally transformed world	Industrial Marketing Management	2020	Digitalization phenomenon		
Fokina, O., Barinov, S	Marketing concepts of customer experience in digital economy	E3S Web of Conferences	2019	Digitalization phenomenon		
Hughes, T., Vafeas, M	Marketing Agency/Client Service-For-Service Provision in an Age of Digital Transformation	Journal of Business-to-Business Marketing	2019	Digitalization phenomenon		
Kaczorowska-Spychalska, D	Shaping consumer behaviour in the fashion industry by interactive communication forms	Fibres and Textiles in Eastern Europe	2018	Social media channels; Mobile technology/smart apps		
Cahyadi, I	Developing Digital Application to Improve Business Process Sustainability in An Indonesian Fast Moving Consumer Goods Company	Journal of Physics: Conference Series	2020	Mobile technology/smart apps	Buying/consumption process	CP
Kim, R.Y	The Impact of COVID-19 on Consumers: Preparing for Digital Sales	IEEE Engineering Management Review	2020	Social media channels; Virtual/augmented Reality		
Venermo, A., Rantala, J., Holopainen, T	From Sales Funnel to Customer Journey	Advances in Intelligent Systems and Computing	2020	Digitalization phenomenon		
Kumar-Singh, A., and Thirumoorthi P	The impact of digital disruption technologies on customer preferences: The case of retail commerce	International Journal of Recent Technology and Engineering	2019	Social media channels; Mobile technology/smart apps; AI; Big Data; Cloud; Virtual/Augmented reality; IoT		
Voronkova, L.P	Virtual Tourism: On the Way to the Digital Economy	IOP Conference Series: Materials Science and Engineering	2018	Virtual/Augmented Reality		
Barnett, J. M	Copyright without creators	Review of Law and Economics	2014	Digitalization phenomenon		
Lin, H., Ouyang, H., Fang, X., Wang, J., Yuan, B., Yang, W	Architecture design and key technologies study of Omnichannel business platform for electric power marketing	Journal of Physics: Conference Series	2020	Digitalization phenomenon	Customer service	CP
Safiullin, M.R., Kurbangalieva, D.L., Elshin, L.A	Supply chain strategy as the instrument of marketing on the example of a platform in the global information space	International Journal of Supply Chain Management	2020	Social media channels		
Lieberman, M	How ‘the new customer buyer’s journey’ is reshaping the way you strategically manage your brand	Journal of Brand Strategy	2019	Digitalization phenomenon		
Polyakov, R.K., Gordeeva, E.A	Industrial enterprises digital transformation in the context of “industry 4.0” growth: Integration features of the vision systems for diagnostics of the food packaging sealing under the conditions of a production line	Advances in Intelligent Systems and Computing	2020	AI; Industry 4.0; Machine Learning	Neuromarketing	CP
Al-Azani, S., El-Alfy, E.-S.M	Enhanced Video Analytics for Sentiment Analysis Based on Fusing Textual, Auditory and Visual Information	IEEE Access	2020	Social media channels	Digital metrics	BPP
Almaslamani, F., Abuhussein, R., Saleet, H., AbuHilal, L., Santarisi, N	Using big data analytics to design an intelligent market basket-case study at sameh mall	International Journal of Engineering Research and Technology	2020	Big Data		
Lin, H., Ouyang, H., Fang, X., Wang, J., Yuan, B., Yang, W	Architecture design and key technologies study of Omnichannel business platform for electric power marketing	Journal of Physics: Conference Series	2020	Digitalization phenomenon		
Melović, B., Jocović, M., Dabić, M., Vulić, T.B., Dudic, B	The impact of digital transformation and digital marketing on the brand promotion, positioning and electronic business in Montenegro	Technology in Society	2020	Social media channels		
Rados, I., Hajnic, M., Rados, I	Digital transformation of monitoring customer behaviour in the cars sales	MIPRO 2020—Proceedings	2020	Digitalization phenomenon		
Safiullin, M.R., Kurbangalieva, D.L., Elshin, L.A	Supply chain strategy as the instrument of marketing on the example of a platform in the global information space	International Journal of Supply Chain Management	2020	Social media channels		
Saravanabhavan, H., Raman, S., Maddulety, K	Value Creation from the Impact of Business Analytics	IFIP Advances in Information and Communication Technology	2020	Digitalization phenomenon		
Sivarajah, U., Irani, Z., Gupta, S., Mahroof, K	Role of big data and social media analytics for business to business sustainability: A participatory web context	Industrial Marketing Management	2020	Big data; social media channels		
Bughin, J., O’Beirne, B., Deakin, J	The anatomy of successful digital transformation: The role of analytics	Applied Marketing Analytics	2019	Digitalization phenomenon		
Garg, N., Kaur, K., Singh, T.G., Singh, M., Jaura, R.K., Sharma, M., Munawar, A., Haque, A	Driving digital transformation using mHealth in clinical research: Bridging the gap to true patient centricity	Plant Archives	2019	Mobile technology/smart apps		
Hausberg, J.P., Liere-Netheler, K., Packmohr, S., Pakura, S., Vogelsang, K	Research streams on digital transformation from a holistic business perspective: a systematic literature review and citation network analysis	Journal of Business Economics	2019	Virtual/ Augmented Reality		
Hsu, P.Y., Huang, C.W., Cheng, M.S., Ko, Y.H., Tsai, C.-H.a, Xu, N	Exploring frequent itemsets in sweltering climates	Communications in Computer and Information Science	2019	Digitalization phenomenon		
Lestari, M.T., Suryana, A., Mulyana, S., Hidayat, M	Why Telco companies in Indonesia using social media monitoring as a way to handle feedback?	Library Philosophy and Practice	2019	Social media channels		
Munz, J., Gaus, C., Doluschitz, R	Analysis of acceptance factors for the use of internet-based information systems in the meat industry	Journal of the Austrian Society of Agricultural Economics	2019	Online collaborative/support platforms/systems; Security protection systems		
Nagano, A	An integrated index towards sustainable digital transformation	Proceedings of the 3rd World Conference on Smart Trends in Systems, Security and Sustainability	2019	Digitalization phenomenon		
Yasynska, N., Fomichenko, I., Voloshyna, O., Byvsheva, L., Ekaterina Krikunenko	Assessment of the level of business readiness for digitalization using marketing and neural network technologies	Innovative Marketing	2019	Digitalization phenomenon		
Majumder, G., Pakray, P., Avendaño, D.E.P	Interpretable Semantic Textual Similarity Using Lexical and Cosine Similarity	Communications in Computer and Information Science	2018	Social media channels		
Papagiannopoulos, N., Lopez, J.F.G	Understanding and predicting passenger behaviours through data analytics	Journal of Airport Management	2018	Big Data		
Caliskan, A., Özkan Özen, Y.D., Ozturkoglu, Y	Digital transformation of traditional marketing business model in new industry era	Journal of Enterprise Information Management	2020	Industry 4.0	Market knowledge	BPP
Endres, H., Helm, R.E., Dowling, M	Linking the types of market knowledge sourcing with sensing capability and revenue growth: Evidence from industrial firms	Industrial Marketing Management	2020	Digitalization phenomenon		
Peter, M.K., Kraft, C., Lindeque, J	Strategic action fields of digital transformation: An exploration of the strategic action fields of Swiss SMEs and large enterprises	Journal of Strategy and Management	2020	IoT		
Rados, I., Hajnic, M., Rados, I	Digital transformation of monitoring customer behaviour in the cars sales	MIPRO 2020—Proceedings	2020	Digitalization phenomenon		
Bohnsack, R., Liesner, M.M	What the hack? A growth hacking taxonomy and practical applications for firms	Business Horizons	2019	Big Data		
Garg, N., Kaur, K., Singh, T.G., Singh, M., Jaura, R.K., Sharma, M., Munawar, A., Haque, A	Driving digital transformation using mHealth in clinical research: Bridging the gap to true patient centricity	Plant Archives	2019	Mobile technology/smart apps		
Hausberg, J.P., Liere-Netheler, K., Packmohr, S., Pakura, S., Vogelsang, K	Research streams on digital transformation from a holistic business perspective: a systematic literature review and citation network analysis	Journal of Business Economics	2019	Virtual/Augmented Reality		
Hsu, P.Y., Huang, C.W., Cheng, M.S., Ko, Y.H., Tsai, C.-H.a, Xu, N	Exploring frequent itemsets in sweltering climates	Communications in Computer and Information Science	2019	Digitalization phenomenon		
Ianenko, M., Ianenko, M., Huhlaev, D., Martynenko, O	Digital transformation of trade: Problems and prospects of marketing activities	IOP Conference Series: Materials Science and Engineering	2019	AI		
Kuimov, V.V., Yushkova, L.V., Scherbenko, E.V., Gunyakov, Y.V	Digital Transformations in the Development of Cooperative Network Interactions	ACM International Conference Proceeding Series	2019	Digitalization phenomenon		
Lestari, M.T., Suryana, A., Mulyana, S., Hidayat, M	Why Telco companies in Indonesia using social media monitoring as a way to handle feedback?	Library Philosophy and Practice	2019	Social media channels		
Munz, J., Gaus, C., Doluschitz, R	Analysis of acceptance factors for the use of internet-based information systems in the meat industry	Journal of the Austrian Society of Agricultural Economics	2019	Online collaborative/support platforms/systems; Security protection systems		
Nosalska, K., Mazurek, G	Marketing principles for Industry 4.0—a conceptual framework	Engineering Management in Production and Services	2019	Industry 4.0		
Yasynska, N., Fomichenko, I., Voloshyna, O., Byvsheva, L., Ekaterina Krikunenko	Assessment of the level of business readiness for digitalization using marketing and neural network technologies	Innovative Marketing	2019	Digitalization phenomenon		
Papagiannopoulos, N., Lopez, J.F.G	Understanding and predicting passenger behaviours through data analytics	Journal of Airport Management	2018	Big Data		
Caliskan, A., Özkan Özen, Y.D., Ozturkoglu, Y	Digital transformation of traditional marketing business model in new industry era	Journal of Enterprise Information Management	2020	Industry 4.0	Communication policy	BPP
Oxoli, D., Terza, V., Cannata, M., Brovelli, M.A	An open IT infrastructure for green tourism management and promotion: The insubri.parks project	International Archives of the Photogrammetry, Remote Sensing and Spatial Information Sciences	2020	Digitalization phenomenon		
Peter, M.K., Kraft, C., Lindeque, J	Strategic action fields of digital transformation: An exploration of the strategic action fields of Swiss SMEs and large enterprises	Journal of Strategy and Management	2020	IoT		
Shkarlet, S., Dubyna, M., Shtyrkhun, K., Verbivska, L	Transformation of the paradigm of the economic entities development in digital economy	WSEAS Transactions on Environment and Development	2020	Digitalization phenomenon		
Yusmarni, Y., Putri, A., Paloma, C., Yusmarni, Y	Marketing performance of Kopi Solok Radjo in industrial revolution 4.0 [a case study of Solok Radjo cooperative in Solok District	IOP Conference Series: Earth and Environmental Science	2020	Industry 4.0; Social media channels; websites/SEO		
Alassani, R., Göretz, J	Product placements by micro and macro influencers on instagram	Lecture Notes in Computer Science	2019	Social media channels		
Ballestar, M.T., Grau-Carles, P., Sainz, J	Predicting customer quality in e-commerce social networks: a machine learning approach	Review of Managerial Science	2019	Websites/SEO; Machine learning		
Dasser, M	Marketing, the change catalyst for digital business transformation: Lessons learned from the modernisation of a B2B marketing organisation	Journal of Brand Strategy	2019	Digitalization phenomenon		
Nosalska, K., Mazurek, G	Marketing principles for Industry 4.0—a conceptual framework	Engineering Management in Production and Services	2019	Industry 4.0		
Cherviakova, V., Cherviakova, T	Value opportunities for automotive manufacturers in conditions of digital transformation of the automotive industry	Journal of Applied Economic Sciences	2018	AI; Mobile technology/smart apps		
Kaczorowska-Spychalska, D	Shaping consumer behaviour in the fashion industry by interactive communication forms	Fibres and Textiles in Eastern Europe	2018	Social media channels; Mobile technology/smart apps		
Bekmurzaev, I., Kurbanov, A., Kurbanov, T., Plotnikov, V., Ushakova, E	Digital technologies of marketing logistics and risks of their implementation in supply chain	IOP Conference Series: Materials Science and Engineering	2020	Industry 4.0	Business process efficiency	BPP
Cahyadi, I	Developing Digital Application to Improve Business Process Sustainability in An Indonesian Fast Moving Consumer Goods Company	Journal of Physics: Conference Series	2020	Mobile technology/smart apps		
Federico, F	A journey of digital marketing transformation: From distributed solo players to embedded digital excellence	Journal of Digital and Social Media Marketing	2020	Digitalization phenomenon		
Li, J., Wu, K., Zhang, B	Analysis and evaluation on the network security defense in power marketing industrial control system	Journal of Physics: Conference Series	2020	IoT; Security protection systems		
Miklosik, A., Evans, N	Impact of Big Data and Machine Learning on Digital Transformation in Marketing: A Literature Review	IEEE Access	2020	Machine Learning; Big Data		
Natorina A	Business optimization in the digital age: Insights and recommendations	Economic Annals-XXI	2020	Websites/SEO		
Sestino, A., Prete, M.I., Piper, L., Guido, G	Internet of Things and Big Data as enablers for business digitalization strategies	Technovation	2020	IoT; Big Data		
Chantayarkul, A., Ayuthaya, S.D.N., Kiattisin, S	The Marketing Strategy for Enhancing the Competitiveness of Local Traditional Stores in Thailand	59th Annual Conference of the Society of Instrument and Control Engineers of Japan	2019	Digitalization phenomenon		
Chehri, A., Jeon, G	The Industrial Internet of Things: Examining How the IIoT Will Improve the Predictive Maintenance	Smart Innovation, Systems and Technologies	2019	Industry 4.0; IoT		
Fiodorov, I., Ochara, N.M	The impact of digital transformation on economic of BRICS countries	CEUR Workshop Proceedings	2019	Digitalization phenomenon		
Graf, M., Peter, M., Gatziu-Grivas, S	Foster strategic orientation in the digital age: A methodic approach for guiding SME to a digital transformation	Lecture Notes in Business Information Processing	2019	Digitalization phenomenon		
Kuimov, V.V., Yushkova, L.V., Scherbenko, E.V., Gunyakov, Y.V	Digital Transformations in the Development of Cooperative Network Interactions	ACM International Conference Proceeding Series	2019	Digitalization phenomenon		
Sargut	Study on the effects of digitisation in small and medium-sized german companies	Quality—Access to Success	2019	Big Data; Machine learning; AI; Chatbots		
Yasynska, N., Fomichenko, I., Voloshyna, O., Byvsheva, L., Ekaterina Krikunenko	Assessment of the level of business readiness for digitalization using marketing and neural network technologies	Innovative Marketing	2019	Digitalization phenomenon		
Papagiannopoulos, N., Lopez, J.F.G	Understanding and predicting passenger behaviours through data analytics	Journal of Airport Management	2018	Big Data		
Ruggieri, R., Savastano, M., Scalingi, A., Bala, D., D’Ascenzo, F	The impact of Digital Platforms on Business Models: An empirical investigation on innovative start-ups	Management and Marketing	2018	Digitalization phenomenon		
Bruskin, S.N., Brezhneva, A.N., Dyakonova, L.P., Kitova, O.V., Savinova, V.M., Danko, T.P., Sekerin, V.D	Business performance management models based on the digital corporation's paradigm	European Research Studies Journal	2017	Online collaborative/support platforms/systems		
Caliskan, A., Özkan Özen, Y.D., Ozturkoglu, Y	Digital transformation of traditional marketing business model in new industry era	Journal of Enterprise Information Management	2020	Industry 4.0	Product policy	BPP
Polyakov, R.K., Gordeeva, E.A	Industrial enterprises digital transformation in the context of “industry 4.0” growth: Integration features of the vision systems for diagnostics of the food packaging sealing under the conditions of a production line	Advances in Intelligent Systems and Computing	2020	AI; Industry 4.0; Machine Learning		
Rados, I., Hajnic, M., Rados, I	Digital transformation of monitoring customer behaviour in the cars sales	MIPRO 2020—Proceedings	2020	Digitalization phenomenon		
Shkarlet, S., Dubyna, M., Shtyrkhun, K., Verbivska, L	Transformation of the paradigm of the economic entities development in digital economy	WSEAS Transactions on Environment and Development	2020	Digitalization phenomenon		
Voipio, V., Elfvengren, K., Korpela, J	In the bowling alley: Acceptance of an intelligent packaging concept in European markets	International Journal of Value Chain Management	2020	IoT		
Bohnsack, R., Liesner, M.M	What the hack? A growth hacking taxonomy and practical applications for firms	Business Horizons	2019	Big Data		
Ianenko, M., Ianenko, M., Huhlaev, D., Martynenko, O	Digital transformation of trade: Problems and prospects of marketing activities	IOP Conference Series: Materials Science and Engineering	2019	AI		
Nosalska, K., Mazurek, G	Marketing principles for Industry 4.0—a conceptual framework	Engineering Management in Production and Services	2019	Industry 4.0		
Ulas, D	Digital Transformation Process and SMEs	Procedia Computer Science	2019	Mobile technology/smart apps; Cloud infrastructure; 3D print; Robotics; Big Data; AI; IoT; Chatbots; Industry 4.0; Virtual/Augmented reality		
Almeida, F., Duarte Santos, J., Augusto Monteiro, J	The Challenges and Opportunities in the Digitalization of Companies in a Post-COVID-19 World	IEEE Engineering Management Review	2020	AI; IoT; Big Data; Robotics	Sales processes	BPP
Venermo, A., Rantala, J., Holopainen, T	From Sales Funnel to Customer Journey	Advances in Intelligent Systems and Computing	2020	Digitalization phenomenon		
Damnjanovic, V	Entry Market Strategy for Weaver Chatbot using the Digital B2B Model	Proceedings—2019 International Conference on Artificial Intelligence: Applications and Innovations	2019	Chatbots		
Dasser, M	Marketing, the change catalyst for digital business transformation: Lessons learned from the modernisation of a B2B marketing organisation	Journal of Brand Strategy	2019	Digitalization phenomenon		
Kumar-Singh, A., and Thirumoorthi P	The impact of digital disruption technologies on customer preferences: The case of retail commerce	International Journal of Recent Technology and Engineering	2019	Social media channels; Mobile technology/smart apps; AI; Big Data; Cloud; Virtual/Augmented reality; IoT		
Seitz, J., Burosch, A	Digital Value Creation	2018 IEEE International Conference on Engineering, Technology and Innovation	2018	Digitalization phenomenon		
Shkarlet, S., Dubyna, M., Shtyrkhun, K., Verbivska, L	Transformation of the paradigm of the economic entities development in digital economy	WSEAS Transactions on Environment and Development	2020	Digitalization phenomenon	Production processes	BPP
Vaganova, O.V., Solovjeva, N.E., Lesovik, R.V., Lesovaya, S.L	Digital transformation of russia’s agricultural sector	Utopia y Praxis Latinoamericana	2020	Digitalization phenomenon		
Chehri, A., Jeon, G	The Industrial Internet of Things: Examining How the IIoT Will Improve the Predictive Maintenance	Smart Innovation, Systems and Technologies	2019	Industry 4.0; IoT		
Del Giorgio, H.R., Mon, A	Usability in ICTs for industry 4.0	Communications in Computer and Information Science	2019	Industry 4.0		
Nosalska, K., Mazurek, G	Marketing principles for Industry 4.0—a conceptual framework	Engineering Management in Production and Services	2019	Industry 4.0		
Andieva, E.Y., Kapelyuhovskaya, A.A	New approaches to digital transformation of petrochemical production	AIP Conference Proceedings	2017	Digitalization phenomenon		
Bekmurzaev, I., Kurbanov, A., Kurbanov, T., Plotnikov, V., Ushakova, E	Digital technologies of marketing logistics and risks of their implementation in supply chain	IOP Conference Series: Materials Science and Engineering	2020	Industry 4.0	Supply chain processes	BPP
Safiullin, M.R., Kurbangalieva, D.L., Elshin, L.A	Supply chain strategy as the instrument of marketing on the example of a platform in the global information space	International Journal of Supply Chain Management	2020	Social media channels		
Sundaram, R., Sharma, R., Shakya, A	Digital transformation of business models: A systematic review of impact on revenue and supply chain	International Journal of Management	2020	Mobile technology; Smart apps		
Voipio, V., Elfvengren, K., Korpela, J	In the bowling alley: Acceptance of an intelligent packaging concept in European markets	International Journal of Value Chain Management	2020	IoT		
Kumar-Singh, A., and Thirumoorthi P	The impact of digital disruption technologies on customer preferences: The case of retail commerce	International Journal of Recent Technology and Engineering	2019	Social media channels; Mobile technology/smart apps; AI; Big Data; Cloud; Virtual/Augmented reality; IoT		
Kazaishvili, A., Khmiadashvili, L	Nurturing M-Learning for Professional Development Through Online Digital Communicational Channels During COVID-19 Pandemic	Lecture Notes in Networks and System	2020	Machine learning; Social media channels	Branding	BPP
Melović, B., Jocović, M., Dabić, M., Vulić, T.B., Dudic, B	The impact of digital transformation and digital marketing on the brand promotion, positioning and electronic business in Montenegro	Technology in Society	2020	Social media channels		
Natorina A	Business optimization in the digital age: Insights and recommendations	Economic Annals-XXI	2020	Websites/SEO		
Rahimian, O	Managing your digital transformation	Proceedings of the Annual Offshore Technology Conference	2020	Digitalization phenomenon		
Dethine, B., Enjolras, M., Monticolo, D	Digitalization and SMEs’ export management: Impacts on resources and capabilities	Technology Innovation Management Review	2020	Digitalization phenomenon	Export market orientation/export performance	BPP
Naglič, A.E., Tominc, P., Logozar, K	The Impact of Industry 4.0 on Export Market Orientation, Market Diversification, and Export Performance	Organizacija	2020	Industry 4.0		
Bollweg, L., Lackes, R., Siepermann, M., Weber, P	Drivers and barriers of the digitalization of local owner operated retail outlets	Journal of Small Business and Entrepreneurship	2020	Digitalization phenomenon	Drivers/barriers/risks of digitalization	BPP
Pirogova, O., Scherbak, M., Maltseva, I	Identification and analysis of risks of digitalization in the field of transport services	IOP Conference Series: Materials Science and Engineering	2020	Digitalization phenomenon		
Agafonova, A.N., Yakhneeva, I.V., Mukhametshina, G.R	Human-Centric Marketing in the Digital Era	Lecture Notes in Networks and System	2020	Digitalization phenomenon	Social responsibility	BPP

11.
